# Intrinsic variables associated with low back pain and lumbar spine injury in fast bowlers in cricket: a systematic review

**DOI:** 10.1186/s13102-023-00732-1

**Published:** 2023-09-20

**Authors:** Patrick Farhart, David Beakley, Ashish Diwan, Rob Duffield, Elizabeth Pickering Rodriguez, Uphar Chamoli, Mark Watsford

**Affiliations:** 1 School of Sport, Exercise and Rehabilitation, Faculty of Health, Human Performance Research Centre, Moore Park Precinct, University of Technology Sydney, Broadway, NSW 2007 Australia; 2 Spine Labs, Discipline of Surgery, St. George and Sutherland Campus of the Clinical School, Faculty of Medicine, University of New South Wales, Kogarah, NSW 2217 Australia; 3 Cricket New South Wales, 161 Silverwater Road, Sydney Olympic Park, Sydney, NSW 2127 Australia; 4 Delhi Capitals, JSW GMR Cricket Private Limited, Bahadurshah Zafar Marg, New Delhi, 110002 India; 5 Deakin University, Burwood Highway, Burwood, VIC 3125 Australia; 6 Spine Service, Department of Orthopaedic Surgery, St. George Hospital Campus, Kogarah, NSW 2217 Australia; 7 School of Biomedical Engineering, Faculty of Engineering and Information Technology, University of Technology Sydney, Broadway, NSW 2007 Australia

**Keywords:** Low back pain, Lumbar spine injury, Fast bowling, Intrinsic variables, Neuromuscular, Kinematics, Kinetics, Technique, Radiology

## Abstract

**Background:**

Lumbar spine injuries in fast bowlers account for the greatest missed playing time in cricket. A range of extrinsic and intrinsic variables are hypothesised to be associated with low back pain and lumbar spine injury in fast bowlers, and an improved understanding of intrinsic variables is necessary as these may alter load tolerance and injury risk associated with fast bowling. This review critically evaluated studies reporting intrinsic variables associated with low back pain and lumbar spine injury in fast bowlers and identified areas for future investigation.

**Methods:**

OVID Medline, EMBASE, SPORTDiscus, CINAHL, Web of Science and SCOPUS databases were last searched on 3 June 2022 to identify studies investigating intrinsic variables associated with low back pain and lumbar spine injury in cricket fast bowlers. Terms relevant to cricket fast bowling, and intrinsic variables associated with lumbar spine injury and low back pain in fast bowlers were searched. 1,503 abstracts were screened, and 118 full‐text articles were appraised to determine whether they met inclusion criteria. Two authors independently screened search results and assessed risk of bias using a modified version of the Quality in Prognostic Studies tool.

**Results:**

Twenty-five studies met the inclusion criteria. Overall, no included studies demonstrated a low risk of bias, two studies were identified as moderate risk, and twenty-three studies were identified as high risk. Conflicting results were reported amongst studies investigating associations of fast bowling kinematics and kinetics, trunk and lumbar anatomical features, anthropometric traits, age, and neuromuscular characteristics with low back pain and lumbar spine injury.

**Conclusion:**

Inconsistencies in results may be related to differences in study design, injury definitions, participant characteristics, measurement parameters, and statistical analyses. Low back pain and lumbar spine injury occurrence in fast bowlers remain high, and this may be due to an absence of low bias studies that have informed recommendations for their prevention. Future research should employ clearly defined injury outcomes, analyse continuous datasets, utilise models that better represent lumbar kinematics and kinetics during fast bowling, and better quantify previous injury, lumbar anatomical features and lumbar maturation.

**Trial registration:**

Open Science Framework 10.17605/OSF.IO/ERKZ2.

**Supplementary Information:**

The online version contains supplementary material available at 10.1186/s13102-023-00732-1.

## Key points

• No included studies were judged to have an overall low risk of bias.

• Inconsistencies in results of studies investigating associations between intrinsic variables and low back pain and lumbar spine injury may be due to differences in study design, injury definitions, participant characteristics, measurement parameters, and statistical analyses.

• Clearly defined injury outcomes, use of continuous datasets, models that better represent lumbar kinematics and kinetics during fast bowling, and improving the quantification of lumbar maturation, previous injury, and lumbar anatomical features represent areas for future research.

**Supplementary Information:**

The online version contains supplementary material available at 10.1186/s13102-023-00732-1.

## Background

Although cricket is a non-contact sport, injury prevalence rates for fast bowlers have been reported to be as high as 20.6% [1] and exceed those reported in football (20%) [2] and rugby (12.0%) [3]. Injury rates of this magnitude may stem from the fast bowling action, which comprises a run-up and straight-arm hurling movement [4], resulting in extreme lumbar motions [5] and torques [6] in the presence of high ground reaction forces (GRF) [7]. These events are postulated to place the lumbar region of fast bowlers at a heightened risk of injury; reflected in high incidences of low back pain (LBP) [8, 9] and lumbar spine injury [1], often manifesting as lumbar intervertebral disc and pars interarticularis abnormalities [10]. Stress fractures of the lumbar spine represent 15% of all missed playing time in cricket [1], and up to 67% of fast bowlers will sustain this injury during their career [11]. Furthermore, stress fractures of the lumbar spine present potentially serious consequences for fast bowlers [11] as they generally cause many months of absence from cricket [12–14], and if not appropriately managed can result in chronic lesions characterised by non-union and recurrence [12, 13].

Multiple risk factors or variables are proposed to interact with one another to contribute to injury susceptibility in athletes [15], and a range of extrinsic and intrinsic variables are hypothesised to be associated with LBP and lumbar spine injury in fast bowlers [16]. Extrinsic variables include bowling workloads [4], match formats [1], and footwear [17]; whereas intrinsic variables may incorporate muscular strength and endurance [18], ranges of motion [18], previous injury [19], biomechanics of the fast bowling technique [5], age [20], and muscle activation [21], morphology [10] and morphometry [22]. Since intrinsic and extrinsic variables do not act in isolation, intrinsic variables may determine the level of risk predisposed [15] to a fast bowler, as elevated risk may cause subsequent exposure to fast bowling to become an inciting event associated with injury [15]. Identifying intrinsic variables is important because they can affect the load tolerance of tissues [23], and an improved understanding of their significance may contribute to the formation of a “cumulative risk profile” [24] for an individual fast bowler. This would represent a holistic assessment of the cumulative influence of intrinsic variables on injury risk with thoughtful consideration of their interaction with one another and with extrinsic variables [24], as this may determine a fast bowler’s capacity to withstand specific training and competition bowling loads, and influence planning and management of the same.

No prior systematic review has specifically reported on intrinsic risk factors associated with LBP and lumbar spine injury in fast bowlers. A narrative review conducted by Johnson et al. [11] reported that excessive shoulder counter rotation (SCR) in adolescents, and excessive contralateral lumbar side-flexion in adults were features of the fast bowling technique that were associated with an increased risk of developing a lumbar stress fracture (LSF). A systematic review examining the association of intrinsic risk factors and successful interventions for LBP in all cricketers (batters, bowlers and wicketkeepers) by Morton et al. [25] identified acute bone stress on MRI scans as a risk factor for LBP and LSF in bowlers. Subsequent systematic reviews examined the association of extrinsic and intrinsic risk factors with all non-contact (lower limb, lumbar, trunk and upper limb) injuries in adult [16] and adolescent [26] fast bowlers. Olivier et al. [16] reported that bowling biomechanics, bowling workload, neuromuscular factors, and previous injury were risk factors for injury, whereas Forrest et al. [26] concluded that injury was associated with bowling biomechanics (excessive lateral trunk flexion and pelvis/hip kinematics), reduced trunk endurance, poor lumbo-pelvic-hip movement control, and early signs of lumbar bone stress on MRI.

The above mentioned systematic reviews [16, 25, 26] presented risk of bias assessments as summary numerical scores using the Downs and Black tool [25], the Joanna Briggs Institute Meta Analysis of Statistics Assessment and Review Instrument [16], and the Newcastle–Ottawa Quality Assessment Scale [26]. The Cochrane Risk of Bias Tool recommends when assessing the risk of bias in studies, it is advisable to select a tool that does not present assessments as summary numerical scores [27], as these have been demonstrated to be poor indicators of study quality [28]. The use of a tool that facilitates a structured assessment, is not based on a scoring system, and is easily adapted for specific needs, is advisable when assessing a study’s risk of bias [28]. Furthermore, previous systematic reviews [16, 25, 26] have not provided detailed information regarding risk of bias evaluations for individual studies, and it has been recommended that researchers should provide supporting statements to justify how risk of bias judgements were reached to minimise subjectivity and maximise consistency of interpretation [29].

The relationship between lumbar spine pathology, missed playing time and LBP in fast bowlers is not straightforward [30], and this is illustrated by asymptomatic fast bowlers presenting with MRI detected pathology [31] as well as high incidences of adolescent fast bowlers presenting with LBP not causing an absence from bowling [9]. Notwithstanding this, lumbar bone stress injuries (LBSI) have been long recognised as a common cause of LBP in fast bowlers [8], and young fast bowlers presenting with LBP contralateral to their bowling arm side represent a high yield population for which an MRI scan provides value for the diagnosis of LBSI [12]. To better understand biases and evidence in the previous literature, it is necessary to examine intrinsic variables that have been associated in studies reporting both LBP and lumbar spine injury, regardless of the presence or absence of symptoms, radiological findings and missed playing time.

Despite extensive research and resources dedicated to identifying intrinsic and extrinsic variables as independent markers for risk of developing LBP and lumbar spine injury in fast bowlers, there has been limited success in predicting and preventing these issues [1]. Whether this lack of predictive insight is related to yet to be identified independent variables, heterogeneity of participant populations [16] or diversity of research methodologies [30] requires further exploration. For example, a recent systematic review reported minimal strength in reported associations between lower back injury and fast bowling workload metrics due to biases within injury and workload measurements in the existent literature [32]. Furthermore, the use of causal inference to classify markers of risk as causal or non-causal may be required, since interventions to prevent injury should be targeted at established causal associations [33].

Given the burden and potential long-term consequences of LBP and LSF in fast bowlers, it is important to better investigate strategies to reduce their incidence [11], and the identified gap in the literature provides an opportunity to conduct a more robust appraisal of intrinsic variables associated with these entities in fast bowlers. The purpose of this review was to critically evaluate studies reporting intrinsic variables that have been associated with LBP and lumbar spine injury in fast bowlers. A further aim was to identify areas for future investigation to assist in the development of effective strategies for the prevention of LBP and lumbar spine injury in fast bowlers.

## Methods

This systematic review was specified a *priori* through protocol registration with the Open Science Framework (10.17605/OSF.IO/ERKZ2, 30 July 2020), and was developed and reported in accordance with the PRISMA-P guidelines for Systematic Reviews [34].

### Data sources and search strategy

For the purposes of data extraction, aspects of a modified PICOC (Population, Intervention, Comparison, Outcome, and Context) framework were applied. Studies evaluating intrinsic variables as risk factors (Intervention) in the development of pain and injuries (Outcome) to the lumbar spine (Context) in cricket fast bowlers (Population) were systematically identified, and the search algorithm was derived from this PICOC framework. Studies published in English or with an available English translation from inception to 27 July 2020 were considered for inclusion into this systematic review. OVID Medline, EMBASE, SPORTDiscus, CINAHL, Web of Science and SCOPUS databases were searched, and the following Boolean search strings were used: (“Cricket” OR “Fast Bowling” OR “Fast Bowler”) AND (“Risk Factor*” OR “Risk” OR “Factor*” OR “Variable” OR “Intrinsic” OR “Age” OR “Adolescent” OR “Young” OR “Adult” OR “Technique” OR “Biomechanic*” OR “Kinematic*” OR “Kinetic*” OR “Strength” OR “Flexibility” OR “Range of motion” OR “Muscle” OR “Asymmetry” OR “Cross Sectional Area” OR “Volume”) AND (“Pain” OR “Injury” OR “Fracture” OR “Stress” OR “Stress Fracture” OR “Stress Reaction” OR “Reaction” OR “Pars interarticularis” OR “Pars” OR “Pedicle” OR “Spondylolysis” OR “Spondylolisthesis” OR “Bone” OR “Oedema” OR “Edema”) AND ( “Low Back” OR “Back” OR “Lumbar” OR “Lumbar Spine”). A detailed search strategy for each database is included in Additional file 1 and this search was repeated on 21 September 2021 and 3 June 2022 to identify new literature. The reference lists of previous systematic reviews were examined to ensure that all potentially relative articles were located, and additional studies were extracted via manual searches of bibliographies, relevant journals, and websites.

### Eligibility criteria

The following criteria were employed to determine the eligibility of literature for inclusion in this review:

#### Types of studies

A range of peer-reviewed journal articles that investigated intrinsic variables associated with the incidence of LBP and lumbar spine injury during fast bowling in cricket were eligible for inclusion. This incorporated both observational (prospective cohort, retrospective cohort, case control, cross-sectional, case series) and interventional (randomised controlled trial, non-randomised controlled trial, quasi-experimental) study designs.

#### Types of participants

Studies included male and female fast bowlers of all age groups and playing levels as participants. A fast bowler was defined as a bowler with a fast run-up, with ball release (BR) speed generally above 100 kph and a wicketkeeper generally standing back from the stumps due to increased BR speed [4].

#### Types of outcome measures

Outcome measures included any lumbar spine condition that resulted in the loss of at least one day of sporting activity, a match time loss injury [1], abnormal radiological features of the lumbar spine, LBSI or LSF, LBP experienced at time of testing or during a study period, a history of LBP, a history of lumbar spine injury, a history of LBSI or a history of LSF.

#### Types of intrinsic variables

Intrinsic variables associated with LBP and lumbar spine injury during fast bowling in cricket that included, but were not limited to, participants’ age, previous injury or pain, biomechanics of the fast bowling technique, muscle strength and endurance, ranges of motion, posture, anthropometric measures, proprioception, bone marrow oedema (BMO) detected on Magnetic Resonance Imaging (MRI), bone mineral density (BMD), bone mineral content (BMC), spinal and trunk muscle thickness, cross sectional area (CSA) and volume.

#### Study selection

All studies identified through search strategies were uploaded into Covidence software [35] and following this, two authors (PF and DB) independently and blindly screened study titles and abstracts to determine their eligibility for inclusion in full text screening. Narrative reviews, systematic reviews, meta-analyses, opinion pieces, non-peer-reviewed articles, conference proceedings, and articles with full-text unavailable were excluded. The full text of eligible studies was then blindly evaluated in an independent manner by the same authors to determine inclusion into the main body of the review. Any disagreements regarding article inclusion were resolved independently by a third author (MW).

#### Data extraction

Data from included studies was extracted by two reviewers (PF and DB) using a modified template based on the Checklist for critical Appraisal and data extraction for systematic Reviews of prediction Modelling Studies (CHARMS) [36]. Extracted data included authors, design, study inference, location, duration, dates, participant information (number, playing level, age, gender, presence of control group), investigated variables, injury outcome, and reported results. Disagreements were resolved through discussion, and if consensus could not be reached, the third reviewer (MW) was consulted.

#### Risk of bias assessment

The methods of risk of bias assessment for included studies were changed from the review protocol registered on the Open Science Framework. Risk of bias was assessed using a version of the Quality in Prognostic Studies (QUIPS) tool [37] modified for this review. QUIPS considers the following six domains to assess potential risk of bias: study participation, study attrition, prognostic factor measurement, outcome measurement, study confounding, and statistical analysis and reporting [37]. Each bias domain contained prompting and consideration items assessed with the terms ‘yes’, ‘partial’, ‘no’ or ‘not reported’; and methodological comments supporting each item’s assessment were recorded. Additional file 2 details defined criteria for the rating of studies. Since responses to individual items may balance or override others, item responses were considered together to assess the risk of bias of each domain, and each domain was rated as having a high, moderate, or low risk of bias.

In line with the Cochrane Risk of Bias Tool for intervention studies [27] and the QUADAS-2 Tool for diagnostic studies [38], computing summated scores for overall study quality using QUIPS is not recommended [27], and this approach was used for this review. Overall study risk of bias was determined as follows: 1) If all domains were low risk, or if one domain was no higher than moderate risk, then a study was classified as low risk, 2) If one or more domains were high risk, or if ≥ 3 domains were moderate risk, then a study was classified as high risk, 3) All studies in between were classified as moderate risk [39]. Two reviewers (PF and DB) assessed risk of bias independently, but were not blinded to authors, title, or journal, and a Quadratic Weighted Kappa score [40] was calculated to determine the level of agreement of individual risk of bias domain judgements between the two reviewers. Disagreements were resolved through discussion, and if consensus could not be reached, the third reviewer (MW) was consulted.

## Results

### Study selection

**Fig. 1:**
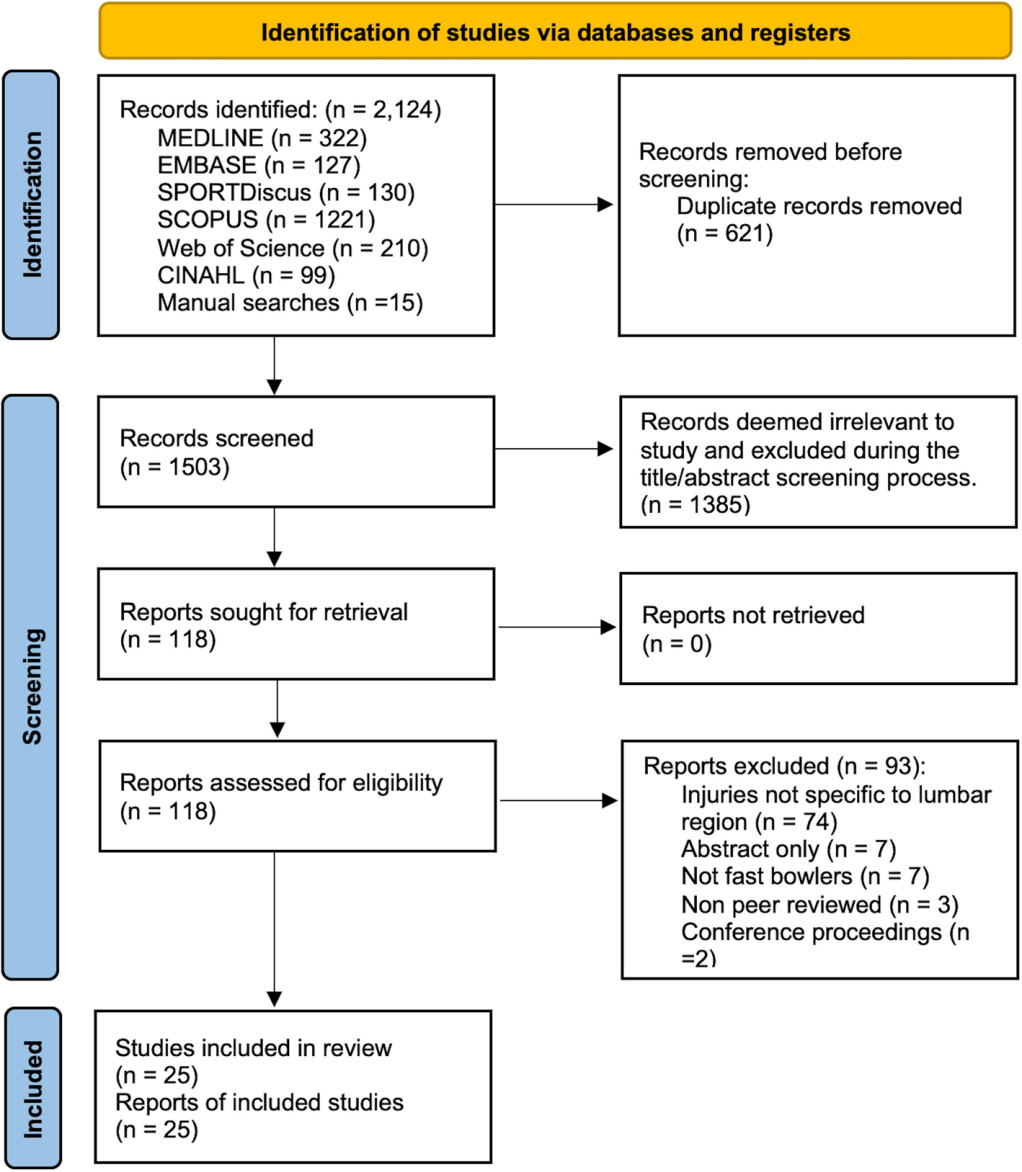
PRISMA flow diagram of search and included studies

As depicted in Fig. 1, database searching generated 2109 studies, and a further 15 were identified following manual searching. 1503 studies remained after duplicates were removed, and 1385 were deemed irrelevant during the title/abstract screening process. Of the 118 studies retained for full text evaluation, 93 were excluded, leaving 25 studies [41–65] for inclusion into this review.

### Risk of bias of studies

**Fig. 2:**
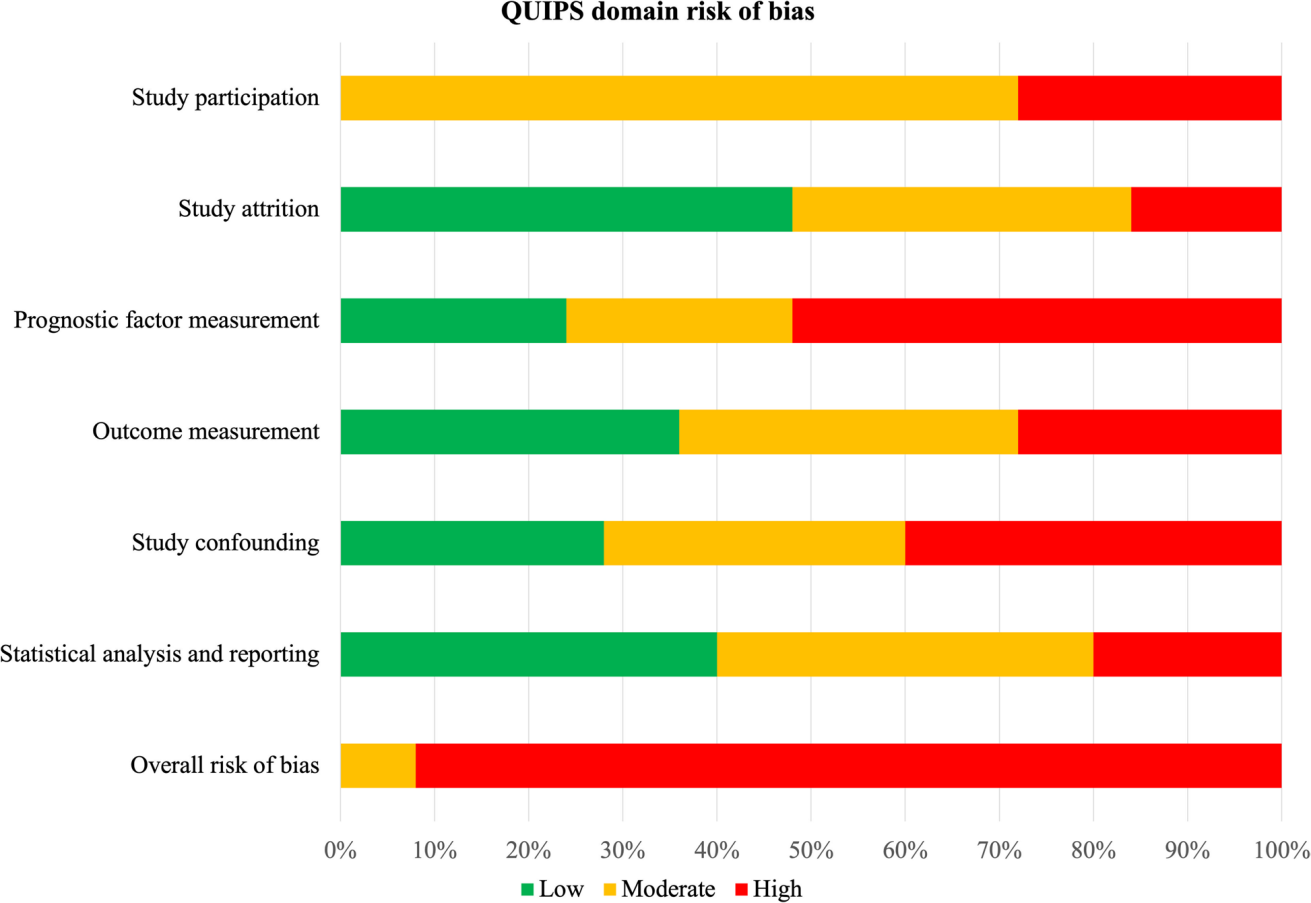
QUIPS risk of bias domain judgements presented as percentages across included studies

The results of risk of bias assessments for each of the six QUIPS domains are presented in Table 1, and Additional file 3 contains detailed information regarding these evaluations. The study screening reviewers (PF and DB) agreed on 124 of 150 items prior to consensus, resulting in a Quadratic Weighted Kappa [54] of 0.84 (95% CI 0.75–0.92). Agreement on domains ranged from 76% (prognostic factor measurement) to 88% (study participation and study confounding). No included studies were categorised as having an overall low risk of bias, two were moderate risk [51, 63], and twenty-three were high risk [41–50, 52–62, 64, 65]. Figure 2 depicts a summary of judgements for each domain’s risk of bias as percentages. Potential sources of bias (moderate risk %, high risk %) were classified as study participation (72%, 28%), study attrition (36%, 16%), prognostic factor measurement (24%, 52%), outcome measurement (36%, 28%), study confounding (32%, 40%), and statistical analysis and reporting (40%, 20%).

**Table 1:** Results of quality assessment of studies using the QUIPS tool

Study	Study participation	Study attrition	Prognostic factor measurement	Outcome measurement	Study confounding	Statistical analysis and reporting	Overall risk of bias
Foster et al. 1989 [42]	High	Moderate	High	High	Moderate	High	High
Elliott et al. 1992 [54]	Moderate	Low	High	High	Moderate	High	High
Hardcastle et al. 1992 [55]	Moderate	Low	High	High	High	High	High
Elliott et al. 1993 [56]	Moderate	Low	High	Moderate	Moderate	Moderate	High
Burnett et al. 1996 [43]	Moderate	High	High	High	High	Moderate	High
Elliott & Khangure 2002 [65]	Moderate	High	High	Moderate	High	Moderate	High
Portus et al. 2004 [60]	Moderate	Moderate	High	Moderate	High	Moderate	High
Engstrom et al. 2007 [44]	Moderate	Low	High	Moderate	Low	Low	High
Stuelcken et al. 2008 [57]	High	Low	Low	Moderate	Low	Moderate	High
Ranson et al. 2010 [45]	High	Moderate	Low	Low	High	Moderate	High
Stuelcken et al. 2010 [58]	High	Low	Moderate	Moderate	High	Low	High
Kountouris et al. 2012 [46]	Moderate	Moderate	High	Low	Low	Moderate	High
Kountouris et al. 2013 [47]	High	Moderate	High	Low	Low	Moderate	High
Olivier et al. 2014 [48]	Moderate	Moderate	Moderate	Moderate	Moderate	Moderate	High
Gray et al. 2016 [62]	Moderate	Low	Low	Low	High	Moderate	High
Bayne et al. 2016 [49]	Moderate	High	Low	High	Low	High	High
Olivier et al. 2017 [50]	Moderate	Moderate	Moderate	Low	High	Low	High
Alway et al. 2019 [51]	Moderate	Low	Low	Low	Moderate	Low	Moderate
Alway et al. 2019 [63]	Moderate	Low	Moderate	Moderate	Moderate	Low	Moderate
Kountouris et al. 2019 [52]	Moderate	Low	Moderate	Low	Moderate	Low	High
Senington et al. 2020 [61]	High	Moderate	Moderate	Moderate	High	Low	High
Alway et al. 2021 [53]	Moderate	Moderate	High	High	High	Low	High
Taylor et al. 2021 [64]	High	Low	High	Low	Moderate	High	High
Sims et al. 2021 [59]	Moderate	Low	High	Low	Low	Low	High
Keylock et al. 2022 [41]	Moderate	High	Low	High	Low	Low	High

### Study characteristics

#### General

Extracted data from included studies [41–65] is presented in Table 2. Studies consisted of thirteen prospective cohort [41–53], five cross-sectional [54–56, 62, 63], three retrospective cohort [57–59], two combined retrospective/prospective cohort [60, 61], retrospective case series [64], and interventional [65] designs. Prospective research was conducted over one cricket season [42, 46–50, 52, 61] or multiple years [41, 44, 45, 51, 53, 60, 65]. Sixteen studies were conducted in Australia [42–44, 46, 47, 49, 52, 54–60, 64, 65], six in the United Kingdom [41, 45, 51, 53, 61, 63] and three in South Africa [48, 50, 62]. Dates of data collection ranging from 1986 [42] through to 2020 [59] were reported in twelve studies [42, 43, 45, 46, 51, 52, 54, 56, 59–61, 65]. A causal association between investigated variables and LBP and lumbar spine injury was implied in twenty-one studies [41–43, 45, 48–56, 58–65].

**Table 2:** Data extraction for included studies (*n* = 25)

Author	Study Design. Inference of study	Participants – Number. Playing level. Age. Gender. Location. Presence of control group. Study dates. Study duration if prospective. Reporting of injury history. Calculation of sample size	Intrinsic variables measured	Lumbar injury outcome	Results
Foster et al. [42]	Prospective cohort. Causality implied	*n* = 82. Potentially high performance from club and school teams. Mean age 16.8 years, age range 15 to 22 years. Male. Australia. No control group included. 1986–87 cricket season. One cricket season duration. No reporting of injury history. No calculation of sample size	Body mass, skinfold levels, anthropometric measurements, hamstring and quadriceps torque, shoulder strength, trunk strength, abdominal muscle endurance, posture, shoulder and hamstring muscle flexibility, aerobic capacity. Bowling kinematics (shoulder and hip alignment at BFC, SCR, BR height). Bowling kinetics (GRF)	CT diagnosed LSF and soft tissue injuries that resulted in a participant missing at least one cricket match.	Stress fracture group bowlers had lower longitudinal foot arch height, greater non-dominant quadriceps torque, higher BR height than non-injured bowlers (*p* < 0.05) and displayed increased SCR (> 40°) between BFC and FFC (non-injured bowlers rotated 15.7°). No association between peak vertical or horizontal GRF during FFC and back injury. Bowlers who sustained a back injury had greater bowling arm shoulder depression and horizontal flexion strength and displayed a shoulder alignment > 240° at BFC (*p* < 0.05). Higher BR position in stress fracture bowlers as a result of more extended front hip and knee joint angles and a more upright trunk during FFC phase
Elliott et al. [54]	Cross sectional. Causality implied	*n* = 20. Western Australian fast bowling development squad. Mean age 17.9 ± 1.6 years. Male. Australia. No control group included. 1989–90 cricket season. No reporting of injury history. No calculation of sample size	Skinfold levels, hamstring/lower back flexibility, abdominal muscular endurance, posture, trunk extension/flexion strength. Bowling kinematics (shoulder and hip alignment at BFC, SCR, BR height). Bowling kinetics (GRF)	Disc degeneration determined by MRI. CT scan diagnosed bone abnormalities categorised as spondylolysis, spondylolisthesis, or pedicle sclerosis	Bowlers with radiological abnormalities had a more front on alignment (no injury 179°, disc abnormality 206°, LSF 197°) at BFC. (*p* < 0.05) and higher degrees of SCR during the delivery stride (no injury 0°, disc abnormality 25°, LSF 12°). Bowlers who adopted a side on technique had the least likelihood of abnormal radiological features, in contrast to those who employed a mixed technique who had the highest rate of radiological abnormalities. LSF bowlers had significantly higher BR height relative to standing height (114% vs 110% in non-injured bowlers) and increased minimum shoulder alignment (LSF 193°, disc 181°, no injury 179°). Age of fast bowlers in injury free group (median 16.4 years) significantly younger than LSF group (median 18.4 years). Sit and reach scores significantly lower in intervertebral disk abnormality group (4.5 cm) than the injury free group (8.0 cm)
Hardcastle et al. [55]	Cross sectional. Causality implied	*n* = 24. Special training in a Western Australian squad. Mean age 17.9 years, age range 16 to 18 years. Male. Australia. Control group 13 batsmen from the same age group. Study dates not described. No reporting of injury history. No calculation of sample size	Kinematics of fast bowling action relating to difference in rotation of the shoulders and the line of the back foot at the time of BFC. Classification of the fast-bowling action whether it was side on, mixed or front on based on shoulder and hip alignment	Disc degeneration determined by MRI. CT scan and radiograph diagnosed bone abnormalities categorised as spondylolysis, spondylolisthesis, or pedicle sclerosis	75% of fast bowlers with a mixed technique experienced LBP. 100% of fast bowlers who rotated > 10° demonstrated a radiological abnormality and 80% of these experienced LBP
Elliott et al. [56]	Cross sectional. Causality implied	*n* = 24. School/club level and showed most potential as determined by match statistics. Mean age 13.7 years. Male. Australia. No reporting of injury history. No control group included. 1991–92 cricket season. No calculation of sample size	Body height, body mass, skinfold levels, hamstring/lower back flexibility, abdominal muscular endurance. Bowling kinematics (shoulder and hip alignment at BFC, SCR and BR height). Bowling kinetics (GRF)	Lumbar intervertebral disc abnormalities (degeneration and bulging) identified by MRI scan	Fast bowlers who counter rotated their shoulders by 30° or more were more likely to sustain a disc abnormality then bowlers who counter rotated their shoulders by 18.9° with *p* = 0.088. Significance level set at 0.1. All abnormal scans recorded from bowlers who used a mixed bowling technique and no bowlers who used side-on, or front-on technique recorded scans with abnormal discs in the lumbosacral region
Burnett et al. [43]	Prospective cohort. Causality implied	*n* = 19. School/club level and showed and showed potential as genuine fast bowlers. Mean age at session 1; 13.6 ± 0.6 years, at session 2; 16.3 ± 0.6 years. Male. Australia. No control group included. Session 1; commencement of the 1991–92 cricket season. Session 2; conclusion of the 1993–94 cricket season. Study duration 3 years. No reporting of injury history. No calculation of sample size	Kinematics of fast bowling action relating to classification as either side on, mixed or front on based on shoulder and hip alignment at BFC. Degree of SCR	Presence of disc degeneration as measured by MRI	Significant increase in occurrence of disc degeneration (21% and 58%, *p* = 0.008) and LBP (5% and 53%, *p* = 0.002) between sessions 1 and 2. The progress of disc degeneration was found to be related to those bowlers who used a mixed bowling action and was seen in 80% of bowlers who used a mixed action during both sessions as opposed to 14% using this technique during one of these sessions only (*p* = 0.015).
Elliott & Khangure [65]	Intervention. Causality implied	*n* = 41. Western Australian Cricket Association fast bowling development squad. Group 1 mean age 13.4. Group 2 mean age 13.2. Male. Australia. No control group included. Group 1; 1997 to 2000. Group 2; 1998 to 2000. Study duration 3 years. No reporting of injury history. No calculation of sample size	Maximum knee flexion angle. Shoulder alignment at BFC, FFC, and at the position of maximum SCR. Classification of fast bowling action whether it was side on, mixed or front on	Abnormal radiological appearance of the intervertebral disk, such as disk degeneration or bulging assessed by MRI. Intervertebral disk degeneration	Bowlers who used a front-on or side-on action recorded significantly lower levels of lumbar disk degeneration when compared with mixed action bowlers (chi-square value 9.5126; *p* = 0.002). In year 1, bowlers who used a front-on or side-on action recorded significantly lower levels of lumbar disk degeneration (12.5%) when compared with mixed action (27%) bowlers. The incidence and progression of lumbar disk degeneration were significantly reduced in parallel with decreased SCR from 35.4° at the commencement of the study to 21.3° in year 4. Progression of the number and/or severity of degeneration only occurred with bowlers who used the mixed action
Portus et al. [60]	Combined retrospective cohort and prospective cohort. Causality implied	*n* = 42. National/international level. Mean age 22.4 ± 3.5 years. Male. Australia. No control group included. Outcomes gathered from 1992 to 2000. No calculation of sample size	Fast bowling technique-related factors; hip-shoulder separation angle at BFC, SCR, hip counter rotation. Classification into side-on, semi-open, front-on and mixed techniques, as well as styles of front lower limb actions during FFC phase	Radiologically diagnosed injuries in the presence of pain classified as either stress fractures of the lumbar spine pars interarticularis or back sprain injuries, including injury to disc, facet joint or ligament	SCR significantly higher in LSF group (41°) than the no trunk injury group (19°) (F = 4.5, *p* = 0.01). Hip counter rotation was highest in the LSF group and lowest in the no injury group. The LSF group had the most front-on shoulder orientation at BFC. At BFC, soft tissue injury group exhibited a larger hip-shoulder separation angle than the no injury group (*p* = 0.03). The LSF group was characterised by a more upright hip joint angle at FFC and at BR, whereas a more flexed knee characterised the non-trunk injured group, but this was statistically non-significant
Engstrom et al. [44]	Prospective cohort. Causality not implied	*n* = 56 at commencement and 51 at conclusion. Club to national level fast bowlers within respective age groups. Age range 13 to 17 years. Male. Australia. Control group of 20 swimmers. Study dates not described. Study duration for fast bowlers 4 years. No reporting of injury history. No calculation of sample size	QL CSA and muscle volume through the lumbar spine and subsequent percent QL asymmetry score for individual bowlers relative to the bowling arm side and swimmers relative to the dominant arm side	Symptomatic pars interarticularis lesions of the lumbar spine diagnosed by MRI.	Significant association between increasing QL asymmetry and the development of symptomatic L4 pars lesions in adolescent fast bowlers with greater QL volumes on the bowling arm side. Increasing QL asymmetry was associated with increased risk. 105% QL asymmetry = 4% risk, 125% QL asymmetry = 58% risk, and 130% QL asymmetry = 78% risk. The area under the nonparametric ROC curve was 0.89 (p ≤ 0.001), indicating excellent discrimination between fast bowlers with and without symptomatic L4 pars lesions, according to percent QL asymmetry
Stuelcken et al. [57]	Retrospective cohort. Causality not implied	*n* = 34; 26 females and 8 males. Females—elite level, Males—Australian Capital Territory representative squad Mean age of females 22.5 ± 4.5 years. Mean age of males 21.5 ± 3 years. Australia. Control group not specifically mentioned but results in females compared to males. Study dates not described. Reporting of injury history in addition to assessment of LBP and study commencement. No calculation of sample size	Range of motion of lumbar flexion, extension, and lateral flexion in standing. Range of motion of hip flexion and hip extension. Trunk extensor endurance	History of LBP if self-reported pain had occurred (1) in the previous 12 months, or (2) at any stage in the bowler’s career and could be verified by records kept by team medical support staff. Episodes of self-reported LBP that did not meet these criteria were disregarded to minimise recall bias	Female fast bowlers with a history of LBP (16.9 ± 3.1°) exhibited a restriction in lumbar lateral flexion range of motion to the bowling arm side compared to those female bowlers with no history of LBP (20.6 ± 7.5°) (*p* = 0.05)
Ranson et al. [45]	Prospective cohort. Causality implied	*n* = 28. ECB Elite Fast Bowling Group, England U19 or senior men’s team. Mean age 19 years, age range 16 to 24 years. Male. United Kingdom. No control group included. 2005—2008. Study duration 2 years. No reporting of injury history. No calculation of sample size	Appearance of lumbar intervertebral discs and BMO in the posterior bony elements on lumbar MRI scans	If bowler missed 7 or more consecutive days of cricket because of LBP. Lumbar stress fracture was recorded if history was corroborated by MRI and/or subsequent CT which confirmed acute bone stress changes associated with partial or complete fracture of the posterior elements of the lumbar spine	Principal finding was that acute stress changes such as oedema and periostitis in the posterior bony elements are related to the subsequent development of a stress fracture. Significant association between appearance of acute bone stress on MRI and subsequent LSF. Of the 15 players who had MRI scans which included presence of acute bone stress on an MRI scan, 11 developed LSF within a mean of 10 weeks from scan time (*p* < 0.001). Of these 15, 9 had no fracture line at scan and they developed LSF at mean of 11 weeks, but 6 did have a fracture line on initial scan and they all went on to develop a LSF within the next 4 weeks. No association found between intervertebral disc degeneration and MRI changes of bone stress (*p* = 0.544) or subsequent LSF (*p* = 0.124)
Stuelcken et al. [58]	Retrospective cohort. Causality implied	*n* = 26. Elite; national/international level. Mean age 22.5 ± 4.5 years. Female. Australia. No control group included. Study dates not described. Reporting of injury history. No calculation of sample size	Pelvis-shoulder separation at BFC. SCR during the delivery stride. Angle of thorax relative to pelvis during the delivery stride. Classification of bowling technique as side-on, front-on, or mixed	LBP history if self-reported pain had occurred in the previous 12 months, or at any stage in the bowler’s career and could be verified by records kept by team medical support staff	The mean maximum lateral flexion range of the thorax relative to the pelvis during the delivery stride was significantly greater for the LBP subgroup compared with the no LBP subgroup (*p* = 0.004), with a large effect size (g = 1.25). The thorax of the LBP subgroup was more laterally flexed away from the bowling arm relative to the pelvis between 73–79% of the delivery stride with upper and lower confidence limits surrounding the difference between the group means being < 0
Kountouris et al. [46]	Prospective cohort. Causality not implied	*n* = 38. Club level adolescents. Mean age 14.9 ± 1.34 years, age range 12 to 17 years. Male. Australia. No control group included. 2002–03 Australian cricket season. Study duration of one cricket season. No reporting of injury history. No calculation of sample size	QL CSA bilaterally at the L3–L4 vertebral levels. BMI	Bowlers who reported LBP and subsequently assessed and diagnosed by a sports physician with either LBSI (lumbar stress fracture or stress reaction of the posterior vertebral element) or soft tissue injury (disc, muscle, or ligament injury)	Significantly higher BMI in bowlers that sustained a LBSI. All 4 asymptomatic participants with radiological evidence of lumbar bone stress at baseline developed symptomatic LBSI. No association between average QL asymmetry for players who sustained lumbar spine injury (soft tissue (12.5%) and bone stress (15.7%) and players who were uninjured (12.4%) (*p* = .537). When participants were grouped as either LBSI (mean QL asymmetry 15.7%) or no LBSI (mean QL asymmetry 12.4%), there was no difference in QL CSA (*p* = .267).
Kountouris et al. [47]	Prospective cohort. Causality not implied	*n* = 23. Identified by respective selection panels as potential national and international players. Mean age 24 ± 3.6; years, age range 19 to 32 years. Male. Australia. No control group included. Study dates not described. Study duration of one cricket season. No reporting of injury history. No calculation of sample size	QL CSA bilaterally between the L2 and L4 vertebral levels. BMI	LBSI; lumbar stress fracture or stress reaction of the posterior vertebral element), which was confirmed radiologically (scintigraphy, MR, or CT), Soft tissue lumbar spine injury (any other injury other than bone stress)	Fast bowlers with no injury had significantly larger asymmetries when compared with those in the soft tissue and bone stress groups (*p* = 0.050). When participants were grouped as either having no injury or any (soft tissue and bone stress) lumbar injury, there was a significant difference between groups (*p* = 0.025). When LBSI group was compared to the two other groups combined, there was no significant difference in mean asymmetry (*p* = 0.949). Four participants (17%) had radiological evidence of lumbar bone stress at baseline, and all went on to develop symptomatic LSF
Olivier et al. [48]	Prospective cohort. Causality implied	*n* = 17. Recruitment derived from questionnaires and telephone calls, but response rate not reported. Amateur premier league club level. Age range 18 to 26 years, but mean age not reported. Male. South Africa. No control group included. Study dates not described. Study duration of one cricket season. Injury history reported. No calculation of sample size	Lumbar proprioception (lumbar reposition error) in the neutral lumbar spine position as well as lumbar positions corresponding to those at FFC and BR of the fast-bowling action	A musculoskeletal condition to the lower back that resulted in loss of at least 1 day of sporting activity or that occurred during a sporting activity that required medical attention, and which forced the bowler to quit the activity.	Of 9 position sense variables tested, lumbar reposition error on 8 associated with previous low back injury; F/E neutral (*p* < 0.01), average neutral (*p* < 0.01), F/E FFC (*p* < 0.02), left/right FFC (*p* < 0.03), average FFC (*P* < 0.01), F/E BR (*p* < 0.01), left/right BR (*p* < 0.03), average BR (*P* < 0.01). Lumbar reposition error on 2 variables associated with low back injury sustained during the period of the study: F/E neutral (*p* < 0.04), average neutral (*p* < 0.03)
Gray et al. [62]	Cross sectional. Causality implied	*n* = 25. Representative cricket at a provincial level. Age range 14 to 18 years. Male. South Africa. No control group included. Study dates not described. No reporting of injury history prior to study commencement. No calculation of sample size	Thickness of the TrA, IO and EO muscles assessed on the dominant and non-dominant side and resultant asymmetry between these measurements	Experiencing LBP for minimum of 6 weeks associated with fast bowling, or bowlers who were currently playing cricket but experienced LBP that caused bowler to miss a match or practice session in the previous 6 weeks	Total combined thickness of TrA, IO and EO on non-dominant side > dominant side in fast bowlers without LBP (post hoc *p* = 0.01) but did not differ between sides for bowlers with LBP (post hoc *p* = 1.0). Greater total thickness in bowlers without pain (3.0 ± 0.4 cm) than with pain (2.4 ± 0.4 cm) on non-dominant side (post hoc—*p* = 0.03), but similar for both groups on dominant side (no pain: 2.5 ± 0.4 cm; pain: 2.5 ± 0.4 cm) (post hoc—*p* = 1.0). Thickness of each abdominal muscle > on non-dominant side in no pain group (post hoc—*p* < 0.001) but did not differ between sides in bowlers with pain (post hoc—*p* = 0.01). Thickness of OI < in pain group than no pain group (post hoc—all *p* = 0.02)
Bayne et al. [49]	Prospective cohort. Causality implied	*n* = 46 who volunteered and underwent initial MRI assessment. 25 included in final analysis. District and/or state junior level. Mean age 15.8 years, age range 14 to 19 years. Male. Australia. No control group included. Study dates not described. Study duration of a 6-month cricket season. No reporting of injury history. No calculation of sample size	Ranges of motion of ankle dorsiflexion, hip internal and external rotation. Foot arch ratio. Trunk extensor endurance, hold times for prone plank and side plank. Tests for lumbopelvic stability, calf endurance, bridge capacity and lower limb movement control. Kinematics of the trunk, pelvis, and lower limbs during the fast-bowling action. Kinetics of the lower limbs and lumbar spine during the fast-bowling action	LBP that affected bowler’s ability to perform in a match, consistent with the consensus of cricket injury. Definition of injury was later expanded to include asymptomatic participants with radiological evidence of lumbar bone stress	Injured bowlers had: lower front hip angle at FFC (46 ± 6° vs 51 ± 6°, t = 2.076, *p* = 0.049), greater thorax lateral flexion at FFC (20 ± 6° vs 15 ± 5°, t = 2.187, *p* = 0.039) and at BR (50 ± 6° vs 40 ± 8°, t = 3.396, *p* = 0.002), increased pelvis rotation beyond front-on at BR (287 ± 11° vs 277 ± 11°, t = 2.408, *p* = 0.024), increased normalised peak flexion (10.5 ± 4.9 Nm/kg/m vs 6.9 ± 2.5 Nm/kg/m,t = 2.292, *p* = 0.036), and lateral flexion lumbar moments (12.5 ± 2.6 Nm/kg/m vs 10.6 ± 1.9 Nm/kg/m,t = 2.079, *p* = 0.049) and peak lateral flexion power (25.8 ± 16.2 W/kg/m vs 14.4 ± 7.7 W/kg/m, t = 2.203,*p* = 0.043), reduced Biering-Sorensen test hold time (103 ± 33 s vs 132 ± 33 s, t = 2.220, *p* = 0.037), increased knee valgus angle during single leg decline squat on dominant (9 ± 3° vs 5 ± 4°, t = 2.299, *p* = 0.031) and non-dominant leg (9 ± 4° vs 6 ± 3°, t = 2.362, *p* = 0.027). A score of 0 on lumbo–pelvic stability test associated with increased risk of low back injury (RR = 1.7, CI 0.78–4.10)
Olivier et al. [50]	Prospective cohort. Causality implied	*n* = 26. From 14 different cricket clubs in Premier league. Mean age 21.8 ± 1.8 years, age range 18 to 26 years. Male. South Africa. No control group included. Study dates not described. Study duration of an 8-month cricket season. Injury history reported. No calculation of sample size	CSA of lumbar MF at the L3, L4, and L5 vertebral levels	A musculoskeletal condition of the lower back that resulted in loss of at least one day of sporting activity or that occurred during a sporting activity that required medical attention, and which forced the bowler to quit the activity	In bowlers who sustained a lower back injury, the nondominant CSA of MF at L3 (*p* = 0.04) and L5 (*p* = .0.04) were smaller than the dominant side MF, however, the percentage difference of the low back injured groups was similar to the non-injured group. No statistically significant differences were found in bowlers with/without asymmetry and bowlers who did/did not sustain lower back injuries (L3 *p* = 0.28; L4 *p* = 0.60; L5 *p* = 1.00)
Alway et al. [51]	Prospective cohort. Causality implied	*n* = 368. English County first or second X1 fast bowlers. Mean age 24.87 ± 6.01 years. Male. United Kingdom. No control group included. 2010 to 2016 England cricket seasons. Study duration of 6 years. No reporting of injury history prior to study commencement. No calculation of sample size	Match bowling workload across all formats of cricket. Age at time of LSF of fast bowlers within this cohort	LSF with diagnosis based on symptomatic presentation and radiological evidence (MRI, CT, or SPECT), which resulted in a player being unavailable for match selection,	74% of LSF occurred in bowlers aged under 25 years, and 56% occurred between the ages 18 and 22. Risk of LSF greatest in bowlers aged 18 to 22, with match incidence at 0.32 per 10 000 deliveries, annual incidence of 4.90 per 100 fast bowlers, and prevalence of 3.21% of squad days. This compared to match incidence of 0.13 per 10 000 deliveries, annual incidence of 2.46 per 100, and prevalence of 1.37% of squad days in the entire cohort of fast bowlers
Alway et al. [63]	Cross sectional. Causality implied	*n* = 23. Selected from existing national senior or developmental squads. Mean age 24.58 ± 3.93 years. Male. United Kingdom. Controls consisted of 14 other cricketers (11 batters, 2 wicketkeepers, 1 spin bowler), 22 rugby players and 20 inactive control participants. Study dates not described. Injury history reported. No calculation of sample size	DEXA measured BMC and BMD of the lumbar spine as a whole (assessed from L1 to L4) and the posterior elements of L3	LSF history determined from England and Wales Cricket Board medical records with any diagnosis confirmed by MRI, CT, or SPECT CT scans	Fast bowlers who never suffered LSF had 3.6% greater BMD in the dominant side of lumbar vertebrae and 1.7% greater BMD in the non-dominant side of lumbar vertebrae compared with those who did suffer LSF, but this was not statistically significant (*p* = 0.08). No significant interaction found between side, vertebra and LSF history on BMC (*p* = 0.34). No significant interaction between side, vertebra, and disc injury history on BMD (*p* = 0.61) or BMC (*p* = 0.77)
Kountouris et al. [52]	Prospective cohort. Causality implied	*n* = 65. Australian junior elite fast bowlers who were selected in an under 17 or under 19 state or territory squad. Mean age 17.3 years, age range 14.7 to 18.8 years. Male. Australia. No control group included. July 2014 to March 2015. Study duration of 8 months. No reporting of injury history. No calculation of sample size	BMO being present or absent through the left and right posterior vertebral arch at each vertebral level from L1 to L5 on sagittal and coronal T2 fat suppressed or short-tau inversion recovery MRI sequences	Development of LBP that caused a participant to be unable to bowl for a period in the study period. Diagnosis of LBSI made with use of clinical judgement and imaging modalities	BMO detected on one or more scans during season associated with 39% of these bowlers suffering a symptomatic LBSI, 37% having persistent BMO but no symptoms, and 24% experiencing a reversal of detected BMO. All bowlers with a symptomatic LBSI had BMO detected at corresponding site of the vertebra in the scan immediately prior to diagnosis. The number of days between the first appearance of BMO on MRI and the player reporting LBSI related symptoms was a mean of 96 days and a median of 112 days. Participants who had BMO detected at any scan at increased risk of BSI (RR = 22.3 (95% CI 1.4—356.6), OR = 36.3 (95% CI 2.1—639.5), with positive predictive value 39%, and negative predictive value 100%
Senington et al. [61]	Combined retrospective cohort and prospective cohort. Causality implied	*n* = 35; 14 seniors and 21 juniors. Participants recruited through coaches from professional county cricket clubs. Mean age senior group 24.1 ± 4.3 years, junior group 16.9 ± 0.7 years. Male. United Kingdom. No control group included. 2015 England cricket season. Duration of prospective portion of study one cricket season. Injury history reported. Sample size was derived from data from [48] with an alpha of 0.05, beta 0.8, effect size of 2 and allocation ratio of 0.75	Spinal orientation at BFC and FFC, spinal range of motion, SCR and hip separation angle. Sacral accelerations. Peak tibia and sacral accelerations along three orthogonal axes with resultant acceleration. Normalised accelerations (to body weight), time-to-peak acceleration, loading rate and time taken to reach peak acceleration	Retrospective: history of LBP. Prospective; any LBP experienced during season. LBP defined as any pain affecting the area of the back inferior to the lower ribs, superior to the inferior gluteal folds and medial to the midaxillary line that impacted on ability to bowl for a minimum of 3 days	No statistically significant results found, but large effect sizes observed. In juniors without LBP history; more thoracic rotation away from direction of delivery (d = 1.3), and a larger range of thoracic rotation between BFC and FFC (d = 0.9). In seniors with LBP history; less peak acceleration around tibial z axis at BFC (d = − 1.5), faster time-to- peak resultant tibial acceleration at FFC (d = − 1.5), and greater thoracolumbar extension at BFC (d = 1.0) In seniors who did not develop LBP; higher time-to- peak resultant tibial acceleration at BFC (d = 1.55), higher tibial loading variables at FFC (d = 0.9), less lumbar extension (d = 1.9) at BFC, more lumbar lateral flexion away from direction of delivery at BFC (d = 1.0), less lumbar extension at FFC (d = 0.9) and less lumbar rotation at FFC (d = 1.3). In seniors who did develop LBP; greater time-to-peak vertical and resultant acceleration at the sacrum (d > 1.6)
Alway et al. [53]	Prospective cohort. Causality implied	*n* = 50. Elite cricket fast bowlers enrolled on an international performance pathway. Mean age 18.9 ± 1.9 years. Male. United Kingdom. No control group included. Study dates not described. Study duration two years. No reporting of injury history. No calculation of sample size	Kinematic parameters of the fast-bowling action; SCR, pelvis-shoulder separation, front leg plant angle, front and rear leg hip and knee angles, lumbopelvic angles, thoracolumbar side flexion and rotation. Kinetic parameters of the fast-bowling action; peak forces, average loading rates and impulse in the vertical and horizontal (braking) directions	LBSI defined as either stress reactions or stress fracture determined from radiological reports the England and Wales Cricket Board injury database. Stress reactions: evidence of bone marrow edema (without fracture line). Acute stress fractures: evidence of incomplete, complete, or multilevel stress fracture accompanied by BMO that suggested the fracture site was active.	At instance of BFC, LBSI bowlers had a more flexed rear hip (d > 0.8, *p* < 0.05) and knee (d > 0.5, *p* < 0.05), less contralateral thoracolumbar side flexion (d > 0.5, *p* < 0.05) and more contralateral thoracolumbar rotation (d > 0.8, *p* < 0.05). At FFC, LBSI bowlers had a more flexed front hip (d > 0.8, *p* < 0.05), more anterior pelvic tilt (d > 0.8, *p* < 0.05), and more extended lumbopelvic angles (d > 0.5, *p* < 0.05). At BR, LBSI bowlers displayed less contralateral thoracolumbar side flexion (d > 0.8, *p* < 0.05). LBSI bowlers had less extension of their front hip and more ipsilateral pelvic drop in transitions between BFC and BR. The best logistic model to predict LBSI included both rear hip angle at BFC and lumbopelvic angle at FFC, correctly classifying 88% of bowlers into injured or non-injured groups. For each 1° increment in rear hip angle at BFC, the odds of having a LBSI was a factor of 0.88 lower, while a 1° increment in lumbopelvic angle at FFC increased the odds of a LBSI by 1.25
Taylor et al. [64]	Retrospective case series. Causality implied	*n* = 38. Elite. Mean age screened group 21.2 ± 3.7 (18.5–24.5) years, self-control group 19.7 (18.5- 24.5) years, matched-control group 21.0 (18.4—24.2) years. Male. Australia. Collected data compared to self-control group (data for same bowler from closest eligible season) and matched-control group (data for a fast bowler closest in age to screened bowler). No reporting of injury history. Study dates not described. No calculation of sample size	Clinically relevant BMO detected at the left and right posterior vertebral arch at each vertebral level (L1-L5)	Symptomatic LBSI that caused missed time from bowling (time loss injury) with MRI identified BMO with or without a fracture	Bowlers with BMO intensity of 2.0 or higher on screening MRI were 1.8 times the risk of sustaining LBSI in the following 12 months compared to bowlers who did not have abnormal BMO detected on screening MRI; RR 1.8 (95% CI 0.6–5.5; *p* = 0.321)
Sims et al. [59]	Retrospective cohort. Causality implied	*n* = 222. Youth fast bowlers in elite pathway programs. Mean age 17.4 ± 1.1 years, age range 15.1–19.7 years. Male. Australia. No control group recruited, but non-injured bowlers acted as controls in multivariate analysis. July 2015 to March 2020. No reporting of injury history. No calculation of sample size	Height, weight, ranges of motion for ankle dorsiflexion, hip internal and external rotation, trunk lateral flexion. Lumbo-pelvic stability, Lumbar extension endurance hold time, single leg balance range, hip abduction and extension strength, aerobic fitness. Age at start of season. Number of balls bowled per day in training and matches. Ranges of SCR and lateral flexion during the fast-bowling action. Bowling speed.	MRI identified LBSI categorised as a stress reaction (bone oedema with no cortical breach) or stress fracture (bone oedema with cortical breach) with a bowler subsequently classified by medical staff as unavailable to train or play	49 of the 222 bowlers sustained a LBSI. Univariate analysis: 1) injured bowlers were younger (*p* = 0.005), taller (*p* = 0.007), performed less efficiently on Star Excursion Balance Test (front foot *p* = 0.006, back foot *p* = 0.005), and on average bowled more days per one week (*p* = 0.009), 4 weeks (*p* = 0.042) and 12 weeks (*p* = 0.008); 2) no difference between injured and non-injured groups in bowling technique variables. Multivariate analysis: risk of LBSI was 2.99 times higher for every year younger in bowlers aged between 15 and 20 years. Bowlers were 1.1 times more likely to be injured for every centimetre taller and 1.1 times more likely for every km/hr faster the ball was bowled. The multivariate model was able to explain 36% of the variance
Keylock et al. [41]	Prospective cohort. Causality implied	*N* = 40. Recruited from professional academies or schools and clubs with well-developed cricket programs. 15.5 ± 1.1 years. Male. United Kingdom. No control group included. Study dates not described. Study duration was 2.26 ± 0.03 years after baseline. No reporting of injury history. No calculation of sample size	Height (cm). Weight (kg). Fat-free mass (kg). Chronological age (years). Skeletal age (years). Skeletal maturity rating. DEXA measured L3 CL BMD (g/cm2), L4 CL BMD (g/cm2), L3 vertebral area (cm2), L4 vertebral area (cm2). CL ankle dorsiflexion (cm). CL ankle dorsiflexion (°). Sit and reach length (cm). Hip internal rotation (°). Hip external rotation (°). Bent knee fall out (°). Straight leg raise (°). Total balls bowled (n. balls). Bowling days per week (n. days). Peak acute workload (n. balls). Peak medium workload (n. balls)	Bowlers who developed symptoms during study with radiologically confirmed LBSI and asymptomatic bowlers who possessed LBSI at the end of the study. LBSI defined as either stress reactions (evidence of BMO without fracture line), or LSF (acute or chronic stress fracture identified by evidence of incomplete or complete stress fracture with BMO)	Chronological age significantly differed at the 0.05 alpha level between prospectively injured and uninjured bowlers (*P* = 0.006), with a large effect size (g = 1.396). Injured bowlers were 1.3 years older at the beginning of the season preceding injury than uninjured bowlers on average although there was little difference in average skeletal age or maturation (g = 0.274 and 0.611, respectively, P ≥ 0.278). 33% of the injured bowlers had delayed maturation, compared to 13% of the uninjured bowlers. Non-significant (P ≥ 0.090) large effect sizes of increased L3 and L4 contralateral BMD (g ≥ 0.812) in LBSI bowlers. Hip internal rotation of the contralateral leg was non significantly 7.2 degrees less in injured fast bowlers compared with uninjured bowlers (32.3 versus 39.5 degrees (g = 0.987)

*BFC* Back foot contact, *SCR* Shoulder counter rotation, *BR* Ball release, *GRF* Ground reaction forces, *CT* Computed Tomography, *LSF* Lumbar Stress Fracture, *FFC* Front foot contact, *MRI* Magnetic Resonance Imaging, *QL* Quadratus Lumborum, *CSA* Cross sectional area, *ROC* Receiver operating characteristic, *LBP* Low Back Pain, *BMI* Body mass index, *F/E* Flexion/extension, *TrA* Transversus abdominis, *IO* Internal Oblique, *EO* External Oblique, *RR* Risk ratio, *OR *Odds Ratio, *MF* Multifidus, *SPECT* Single Photon Emission Computerised Tomography, *DEXA* Dual Energy X-ray Absorptiometry, *BMC* Bone mineral content, *BMD* Bone mineral density, *BMO* Bone marrow oedema, *CL* Contralateral, *CI* Confidence Interval

#### Participants

Mean chronological age was reported in all studies [41–65], ranging from 13.2 [65] to 24.9 years [51], and four studies included age-matched control groups [44, 51, 55, 64]. Only two studies did not contain exclusively male participants, with one including females only [58] and another females and males [57]. Studies recruited elite level adults [45, 47, 51, 53, 57, 58, 60, 61, 63, 64], elite level adolescents [46, 54, 55, 59, 61, 62, 65], club and/or school level adolescents [42, 43, 46, 56], adolescents at varying skill levels [41, 44, 49], and club level adults [48, 50]. At commencement of seventeen studies [41–50, 52–54, 56, 61, 64, 65], bowlers were deemed fit to bowl and had no knowledge of abnormal radiological features, but may have experienced LBP in two of these [43, 56]. Six studies [55, 57, 58, 60, 62, 63] included bowlers with and without injury outcomes at commencement, and two studies [51, 59] exhibited incomplete disclosure of injury status at commencement.

#### Injury outcomes

Eleven studies [42, 44–47, 51, 52, 59, 60, 63, 64] reported injury if a combination of lumbar symptoms, abnormal radiology and missed playing time were present. LBSI was defined across studies [46, 47, 52, 59, 64] as an MRI confirmed stress reaction (BMO with no cortical breach) or stress fracture (BMO with cortical breach) [52]. LSF [42, 45, 51, 60, 63] and pars interarticularis lesions [44] were reported as partial or complete fractures [45], or with no description of cortical breach presence [42, 44, 51, 60, 63]. Soft tissue injuries [42, 46, 47, 60] were reported without elucidation [42], or defined as disc, muscle, or ligament injury [46], injuries other than bone stress [47], or categorised as back sprain (disc, facet joint or ligament) injuries [60]. Six studies described LBP in the absence of radiology findings as an injury outcome [48, 50, 57, 58, 61, 62]; derived from questionnaires investigating previous LBP occurrence [48, 50, 61, 62], self-reported LBP in the previous 12 months [57, 58], recording of LBP during a study [48, 50, 61], or self-reported LBP verified by medical records [57, 58].

Studies describing injury in the absence of symptoms [43, 53–56, 65] reported LBSI determined from MRI reports [53], disc degeneration determined by MRI features [43, 54–56, 65], and Computed Tomography (CT) diagnosed bone abnormalities categorised as spondylolysis, spondylolisthesis, or pedicle sclerosis [54, 55]. Two studies [41, 49] categorised injury in bowlers with and without symptoms; with one defining injury as LBP affecting a bowler’s ability to perform in a match and expanded this to include asymptomatic bowlers with MRI detected lumbar bone stress [49]. Subsequent research categorised injury as symptomatic LBSI causing missed playing time as well as MRI reported LBSI in asymptomatic bowlers [41].

#### Intrinsic variables measured

A summary of intrinsic variables reported in included studies is contained in Table 3 and definitions for these variables are contained in Additional file 4. Twelve studies investigated bowling technique biomechanical variables [42, 43, 49, 53–56, 58–61, 65] utilising two-dimensional (2-D) motion analysis [42, 43, 54–56, 65], three-dimensional (3-D) motion analysis [49, 53, 58, 60], both 2-D and 3-D motion analysis [59], and inertial measurement units (IMUs) [61]. Four studies assessed trunk kinematics [55, 58, 59, 61]; whereas eight measured both trunk and lower limb kinematics[42, 43, 49, 53, 54, 56, 60, 65]. Seven studies quantified kinetics [42, 49, 53, 54, 56, 60, 61]; six measured GRF with force plates/platforms [42, 49, 53, 54, 56, 60], and IMUs captured sacral and tibial rates of loading and impacts in another [61]. Biomechanical testing environments were laboratory based [42, 43, 53, 54, 56, 58, 60, 65], outdoors [61], laboratory and netted [59], and not specified in two studies [43, 55]. Discrete point analysis techniques were used to identify kinematic and kinetic variables in all biomechanical studies [42, 43, 49, 53–56, 58–61, 65]. Descriptive variables collected included BR height [42, 54, 56], BR speed [53, 54, 56, 59], approach velocity [42, 49, 53, 54], delivery stride length [54, 56], and delivery stride alignment [54].

**Table 3:** Summary of intrinsic variables reported in included studies

Categories of variables	Intrinsic variables measured	Studies
Biomechanics of fast bowling technique	Shoulder and hip alignment, SCR	[42, 43, 49, 53–56, 58–61, 65]
	Lower limb kinematics	[42, 43, 49, 53, 54, 56, 60, 65]
	Trunk and lumbar rotation	[49, 53, 58, 61]
	Trunk and lumbar flexion/extension	[49, 53, 58, 61]
	Trunk and lumbar lateral flexion	[49, 53, 58, 59, 61]
	Ground reaction forces	[49, 52–54, 56, 60]
	Tibial and sacral loading	[61]
	Lumbo-pelvic kinetics	[49]
	Ball release height	[42, 54, 56]
	Ball release speed	[53, 54, 56, 59]
	Approach velocity	[42, 49, 53, 54, 56]
	Delivery stride length	[54, 56]
	Delivery stride alignment	[54]
Trunk and lumbar anatomical characteristics	Quadratus Lumborum asymmetry	[44, 46, 47]
	Multifidus CSA	[50]
	Abdominal muscle thickness	[62]
	Lumbar vertebrae BMO presence	[45, 52, 64]
	Lumbar disc degeneration	[45]
	Lumbar spine BMC/BMD	[41, 63]
Age	Chronological age	[41, 51, 54, 59]
	Skeletal age	[41]
Injury history	Previous LBP or lumbar injury	[48, 50, 61]
Muscle strength, endurance, and function	Trunk flexion/extension strength	[42, 54]
	Hip abduction/extension strength	[59]
	Hamstring/quadriceps strength	[42]
	Calf/single leg bridge capacity	[49]
	Shoulder depression/horizontal flexion strength	[42]
	Abdominal sit ups	[42, 54, 56]
	Trunk plank capacity	[49]
	Trunk extensor endurance	[49, 57, 59]
Range of motion	Sit-and-reach	[41, 42, 54]
	Shoulder flexibility	[42]
	Lumbar flexion/extension	[57]
	Lumbar lateral flexion	[57, 59]
	Straight leg raise	[41, 57]
	Hip extension	[57]
	Hip external/internal rotation	[41, 49, 59]
	Bent knee fall-out	[41]
	Ankle dorsiflexion	[41, 49, 59]
Physical characteristics	Skinfold levels	[42, 54, 56]
	Foot arch features	[42, 49, 54]
	Body mass	[42, 54, 56, 59]
	Fat free mass	[41]
	Body mass index	[46, 47]
	Segment anthropometrics	[42]
	Posture	[42, 54]
	Height	[41, 59]
	Aerobic capacity	[42, 59]
Neuromuscular control	Lumbar Reposition Error	[48]
	Single leg decline squat	[49]
	Lumbopelvic stability	[49, 59]
	Star Excursion Balance	[59]

*SCR* Shoulder Counter Rotation, *CSA* Cross sectional area, *BMO* Bone marrow oedema, *BMC* Bone mineral content, *BMD* Bone mineral density, *LBP* Low Back Pain

Ten studies applied radiological investigations to assess trunk and lumbar anatomical characteristics [41, 44–47, 50, 52, 62–64]. MRI was used to quantify Quadratus Lumborum (QL) muscle asymmetry [44, 46, 47], whereas Ultrasound (US) was employed to measure Multifidus CSA [50] as well as Transversus Abdominis, Internal Oblique and External Oblique thickness [62]. Three studies utilised MRI to investigate BMO presence in lumbar vertebrae and its association with future injury [45, 52, 64], and one examined lumbar intervertebral disc degeneration for this purpose [45]. Dual Energy X-ray Absorptiometry (DEXA) was used to assess lumbar spine BMC and BMD [41, 63], vertebral body area [41], and skeletal age was assessed with DEXA of the left hand [41] and analysed by the Tanner and Whitehouse Three method [66]. Chronological age [41, 51, 54, 59] and a history of LBP or lumbar injury [48, 50, 61] were investigated as variables that may be associated with future LBP and lumbar spine injury.

Strength measures included testing of maximal trunk flexion and extension strength [42, 54], maximal hip abduction and extension strength [59], isokinetic hamstring and quadriceps muscle strength [42], calf muscle and single leg bridge capacity [49], and shoulder depression/horizontal flexion strength [42]. Abdominal muscular function was assessed with prone and side plank tests [49], and a 60 s sit up test [42, 54, 56], whereas trunk extensor endurance was assessed with the Biering-Sorensen test [49, 57, 59].

Range of motion assessments included sit-and-reach [41, 42, 54], shoulder flexibility [42], lumbar flexion [57], extension [57], and lateral flexion in standing [57, 59], passive straight leg raise [41, 57], modified Thomas test for hip extension [57], hip external and internal rotation [41, 49, 59], bent knee fall-out for hip flexibility [41], and ankle dorsiflexion with lunge testing [41, 49, 59]. Physical characteristics including skinfold levels [42, 54, 56], foot arch features [42, 49, 54], body mass [42, 54, 56, 59], fat free mass [41], body mass index (BMI) [46, 47], segment anthropometrics [42], posture [42, 54], standing height [41, 59], and aerobic capacity [42, 59] were assessed. Lumbar Reposition Error (LRE) in neutral spine and fast bowling specific positions [48], a single leg decline squat test [49], a lumbopelvic stability test [49, 59], and a Star Excursion Balance Test (SEBT) [59] were employed as neuromuscular control assessments.

### Results of individual studies

#### Age

Prospective research reported an association between LSF and younger chronological age in professional fast bowlers (mean age 24.87); as 74% of LSFs occurred in bowlers aged under 25 years, with an annual incidence of 4.90 LSFs per 100 bowlers aged 18 to 22 compared to 2.46 across the entire cohort [51]. A range of associations between age and injury have been reported in adolescent cohorts [41, 54, 59]; as LBSI risk was 2.99 times higher for each year younger in bowlers aged 15 to 20 years [59], and bowlers with LBSI were older than injury free bowlers (median 18.4 vs 16.4 years) [54], whilst injured bowlers were 1.3 years older than uninjured bowlers, despite no difference in average skeletal age or maturation [41].

#### Previous lumbar injury

No associations were reported between incidence of previous and future lumbar injury in two studies [48, 50]. However, previous LBP is potentially a good prognostic indicator for recurrent injury, as all bowlers who developed LBP during a prospective study reported previous LBP at study commencement [61].

#### BMC/BMD

Non-significant associations were reported in two studies investigating relationships between lumbar BMD and injury [41, 63]. In a cross-sectional study, adult bowlers with previous LSF had 3.6% and 1.7% lower BMD in dominant and non-dominant sides of lumbar vertebrae respectively compared to bowlers without LSF history [63]. In contrast, prospective research reported greater non-dominant side BMD of the L3 and L4 vertebrae by way of larger effect sizes in adolescents who developed LBSI [41].

#### Trunk and lumbar muscle morphology and morphometry

Asymmetries presenting as increased QL volume on the bowling arm side have been associated with symptomatic L4 pars lesion development [44] in adolescents. However, subsequent research reported no significant association between QL CSA asymmetry and future LBSI in adolescents [46] and adults [47].

Whilst Multifidus CSA on the contralateral side to the bowling arm at L3 and L5 was reported to be smaller in adults who developed a lumbar injury, no associations were reported for between-sides percentage differences or asymmetry [50]. The individual and total combined thicknesses of three abdominal muscles (Transversus Abdominus, Internal Oblique, External Oblique) on the side opposite to the bowling arm were reported to be greater in pain-free adolescents compared to those experiencing LBP [62].

#### Presence of BMO

In elite bowlers, BMO at baseline MRI was associated with symptomatic LSF development at a mean of 10 weeks from scan time [45], and bowlers with cortical breach at baseline developed symptoms within 4 weeks, compared to a mean of 11 weeks for those with no cortical breach [45]. In adolescents who underwent six MRI scans throughout an eight-month cricket season, all participants who sustained a symptomatic LBSI had corresponding site BMO detected in the scan immediately prior to diagnosis, with a mean of 96 days between initial BMO appearance and symptom reporting [52]. When results of all scans were pooled, a relative risk of 22.3 (95% CI 1.4 to 256.6) was reported for detected BMO leading to a symptomatic LBSI; with a Positive Predictive Value of 39.5 and a Negative Predictive Value of 100 [52]. Elite bowlers nominated for screening by their medical team with a BMO intensity ratio of ≥ 2.0 on MRI were reportedly at 1.8 times greater risk of sustaining LBSI in the following 12 months compared to bowlers with no abnormal BMO detected; with a median of 258 days between scan time and injury diagnosis [64].

#### Biomechanics of fast bowling

Biomechanical studies were prospective [42, 43, 49, 53], cross-sectional [54–56], retrospective [58, 59], combined prospective/retrospective [60, 61], and interventional [65] in nature. In predominantly adolescent fast bowlers, greater BR height was associated with lower back injuries [42] and lumbar bone abnormalities [54], but not disc degeneration [56]. The association of BR speed and lumbar spine injury is inconsistent, as studies in adults [53] and adolescents [54, 56] reported no associations, whereas subsequent research reported LBSI risk increasing 1.1 times for every km/h faster BR speed in adolescents [59].

Reported associations between shoulder alignment and lumbar spine injury are inconsistent. A front-on shoulder alignment at back foot contact (BFC) has been associated with LSF in adults [60], and with LSF [54], lumbar disc [54] and lower back [42] injuries in adolescents. Interestingly, other studies have reported no association with disc degeneration in adolescents [56] or lower back injury in adolescent [49, 61] or adult [53, 61] bowlers.

A front-on shoulder alignment at BFC has been linked to increased SCR, which is the change between shoulder alignment at BFC and the minimum shoulder alignment during the delivery stride [60], however, the association of SCR with lumbar spine injury is inconsistent. SCR has been associated with LSF [42], disc abnormalities [56, 65], and radiological abnormalities [54] in adolescents, and LSF in adults [60]. Subsequent studies however reported no association between SCR and LBP history in elite females [58], adolescents and adults [61]; or future lumbar spine injury in adolescent [49, 59, 61] and adult cohorts [53, 61].

In adults, associations between increased hip counter rotation and hip-shoulder separation (HSS) at BFC with LSF and back sprain injury respectively were reported; and a large HSS angle at BFC along with high SCR define a mixed technique [60]. Higher rates of radiological abnormalities [54], disc degeneration [43, 56, 65], and LBP [55] in adolescents, and lower back injuries in adults [60] have been associated with a mixed technique. However, other studies reported no such associations with LBP history in elite females [58] or lumbar spine injury in adolescent [49, 59, 61] or adult fast bowlers [53, 61].

Conflicting results have been reported for associations between lateral flexion away from the bowling arm and lumbar spine injury [49, 53, 58, 59, 61]. Thorax lateral flexion was greater during the delivery stride in elite females with LBP history [58], and at front foot contact (FFC) and BR in adolescents who developed lumbar spine injury [49]. Injured elite males had less thoracolumbar lateral flexion at BFC and BR, and a medium effect size for increased lumbopelvic lateral flexion at BR [53]; whilst no association was reported for lumbopelvic lateral flexion range between FFC and BR and injury elsewhere [49]. Further to this, large effect sizes have been reported for increased lumbar lateral flexion at BFC in adults who did not develop LBP [61], and a recent 2-D study demonstrated no relationship between trunk lateral flexion and LBSI in adolescents [59].

Thorax flexion and extension relative to the pelvis did not have any association with LBP history in elite females [58]. Lumbopelvic flexion/extension has an inconsistent relationship with injury; as this was not associated with lower back injury in adolescents [49], but in adults each 1° increment in the lumbopelvic extension angle at FFC increased the odds of LBSI by 1.25 [53]. Furthermore, large effect sizes have been reported for greater thoracolumbar extension at BFC in adults with LBP history [61], and for reduced lumbar extension at BFC and FFC in adults who did not develop LBP [61].

Associations between LBP and lumbar spine injury and rotation metrics are inconsistent [49, 53, 58, 61]. Neither thorax rotation relative to the pelvis [58] or lumbopelvic rotational range [49] were associated with LBP history in elite females [58] or lower back injury in adolescents [49]. However, subsequent research reported that injured adults had increased thoracolumbar rotation away from the bowling arm at the instance of BFC [53]. In adolescents without LBP history, large effect sizes were reported for increased thoracic rotation away from the bowling arm at BFC and an increased range of thoracic rotation between BFC and FFC [61]. In the same study, a large effect size for reduced lumbar rotation at FFC was observed in adults who did not develop LBP [61].

Pelvis-shoulder separation at BFC had no association with LBP history in elite females [58] or LBSI in adults [53]. Increased pelvis rotation beyond front-on at BR was reported in adolescents who developed lower back injury [49] and increased anterior pelvic tilt at FFC and increased ipsilateral pelvic drop in transitions between BFC and BR were reported in adults who sustained LBSI [53].

Injured adults were reported to have more flexed rear hip and knee angles at the instance of BFC, and the degree of rear hip flexion was reported to categorise LBSI in 76% of bowlers, with odds of injury reduced by a factor of 0.88 for each 1° increment in rear hip extension [53]. Reduced front hip flexion during FFC has been associated with LSF [42] and lower back injury [49] in adolescents, and LSF in adults [60]. Conversely, adults with LBSI were reported to have more flexed front hip angles at FFC and possess less front hip extension in transitions between BFC and BR [53].

The relevance of front knee angles to injury [42, 49, 53, 56, 60, 65] has also yielded conflicting results. Whilst predominantly adolescent bowlers who developed LSF tended to have increased front knee extension [42], and non-injured adults displayed increased front knee flexion during FFC [60], other studies have reported no association of front knee flexion during FFC and at BR with lumbar spine injury in adolescents [49, 56, 65] and adults [53]. No associations were reported in adults between LBSI and front foot and front leg plant angles at the instant of FFC [53].

GRF magnitudes at BFC and FFC have not been significantly associated with lumbar spine injury [42, 49, 53, 54, 56, 60]; however, adults who experienced LSF displayed tendencies for higher vertical GRF at BFC, and faster rates of peak braking and vertical force development during FFC [60]. Large effect sizes were reported for reduced peak tibial Z axis acceleration and faster time to peak resultant tibial acceleration at FFC in adults with LBP history [61], whereas those who did not develop LBP experienced higher time to peak resultant tibial acceleration at BFC and higher tibial loading variables at FFC [61]. Large effect sizes were reported for greater time-to-peak vertical and resultant acceleration at the sacrum in adults who developed LBP [61]. Injured adolescents displayed increased peak lateral flexion power and normalised peak flexion and lateral flexion lumbar moments in comparison to non-injured counterparts [49].

#### Physical characteristics

Whilst an association was reported in adolescents between lower longitudinal foot arch height and LSF [42], other research reported no such relationship with lower back injury [49] or radiological abnormalities [54]. An association between higher BMI and LBSI was reported in adolescents [46], but not in adults [47]; and adolescents were reportedly 1.1 times more likely to sustain LBSI for every centimetre taller in standing height [59].

Range of motion assessments have yielded conflicting results, with sit and reach scores being lower in adolescents with intervertebral disc abnormalities [54], but not in those with disc degeneration [56] or LBSI [41]. Elite females with LBP history had reduced lumbar lateral flexion range to the bowling arm side [57]; however, lumbar lateral flexion range was not associated with LBSI in in adolescent males [59]. Hip internal rotation of the non-dominant leg was reported to be 7.2° less in adolescents who sustained an LBSI, however this was not statistically significant [41].

Analyses of muscle strength and endurance have reported varying associations with lumbar spine injury incidence [42, 49, 54, 56, 57, 59]. Greater front leg quadriceps and bowling arm shoulder depression and horizontal flexion strength have been reported in predominantly adolescent bowlers with LSF and lumbar injuries respectively [42]. Whilst reduced trunk extensor endurance was reported in adolescents who developed lower back injury [49]; other research reported no association between this and LBP history in elite females [57], or LBSI in adolescents [59].

Reduced performance of the lumbopelvic stability test in adolescents was reported to be associated with a 1.7 times increased risk of lower back injury in one study [49], but not related to LBSI in another [59]. In adolescents, increased knee valgus angle during a single leg decline squat on both legs was reportedly associated with increased lower back injury risk [49]. Whilst SEBT distance was deemed not to be significant in multivariate analysis, adolescents who sustained LBSI performed less efficiently on this test [59]. LRE in two neutral spine and six fast bowling specific positions was associated with LBP history, and LRE in two neutral positions was associated with a future lower back injury [48].

## Discussion

This systematic review reported on intrinsic factors associated with LBP and lumbar spine injury in fast bowlers. Conflicting results were reported amongst studies investigating fast bowling biomechanics [42, 43, 49, 53–56, 58–61, 65], trunk and lumbar muscle asymmetries [44, 46, 47, 50, 62], anthropometric characteristics [42, 46, 47, 49, 54, 56, 59], muscle strength and endurance [42, 54, 56, 57, 59, 63], ranges of motion [41, 42, 49, 54, 57, 59], neuromuscular control [48, 49, 59], age [41, 51, 54, 59], and lumbar BMD [41, 63]; whereas more consistent results were described when reporting lumbar vertebra BMO and its association with subsequent LBSI [45, 52, 64]. Inconsistencies in results may reflect differences in study design, injury definitions, participant characteristics, measurement parameters, and statistical analyses.

### Risk of bias

Risk of bias appraisal is essential as increased bias affects the internal validity of studies [37, 67] that may inform strategies for injury prevention in fast bowlers. Aspects of bias relating to assessed QUIPS domains and how these inform directions for future research will be discussed herein, particularly given the high overall risk of bias in many studies evident in Fig. 2.

#### Participation bias

Eighteen studies [41, 43, 44, 46, 48–56, 59, 60, 62, 63, 65] were classified as having a moderate risk of participation bias, and the remaining seven [42, 45, 47, 57, 58, 61, 64] were rated as high risk. Studies with the lowest sample sizes [43, 45, 47–50, 54–58, 62, 63] may have been inadequately powered [68], and this possibly contributed to the low replicability of results from included studies. The non-reporting of recruitment methodologies [44, 46, 49, 62, 63] and response rates to recruitment [48, 50] may reflect an absence of eligible participants and reduced study representativeness. Volunteer bias [67] may have occurred in studies that recruited higher proportions of injured participants [53, 62] and in research not reporting injury history, which was described in only four included studies [44, 48, 50, 61]. Selection bias [67] may have occurred in studies exhibiting targeted recruitment of participants by coaches [42, 45, 57, 58, 61], selectors [47], and medical staff [64], and in another excluding 15 asymptomatic bowlers at study commencement due to MRI findings [49]. Survivor bias may have existed in professional cohorts [47, 51, 57, 58, 60, 63], as previously injured bowlers may have become slow bowlers, specialist batters, or no longer be playing cricket at the elite level [57].

The transparent reporting of recruitment across adolescent, adult, amateur and professional fast bowling cohorts is an important first step in reducing participation bias. Multifaceted recruitment methods incorporating personal contact, social media initiatives, and partnerships with stakeholders based on education and dissemination of research results will improve power and representativeness of future research [68], and subsequent random selection of participants from pre-established cohorts can reduce volunteer bias [69]. Sample size calculations were conducted for only one included study [61], and these should be considered in future studies incorporating accuracy in parameter estimation, sequential testing, and Bayesian models, as these approaches may improve the precision of measurements and detection of small effects [70]. Moreover, the establishment of international collaborations to acquire datasets of sufficient sample size and heterogeneity may improve the external validity of future research [71].

#### Attrition bias

Apart from lowering study power, attrition threatens both external and internal validity of results [72], as participants who are most impaired are more likely to be lost to follow-up [73]. A moderate risk of attrition bias was judged to be present in the following study designs that did not report participants potentially lost to follow-up; prospective with one cricket season length [42, 46–48, 50], prospective in elite environments over multiple seasons [45, 53], and combined retrospective/prospective [60, 61]. Prospective research not reporting dropouts in non-elite environments over multiple years [43, 65] was classified as high risk, as was a study not describing reasons for six participants missing from initial recruitment [49], and another with a reported dropout rate of 45% [41].

Reducing attrition using matching retention strategies to samples prior to study implementation, including careful consideration of unintended burden for participants [74] is required in future prospective research. In studies where attrition has occurred, bias can be reduced with reporting of sample size at each data collection point, reasons associated with loss of participants, and statistical analysis of dropouts versus those remaining across demographic data, pre-test responses, and variables particular to studies [72].

#### Prognostic factor measurement bias

Six studies [48, 50, 52, 58, 61, 63] were classified as having a moderate risk of prognostic factor measurement bias, and thirteen were rated as high risk [42–44, 46, 47, 53–56, 59, 60, 64, 65]. The use of single trials [42, 43, 48, 53–56, 60, 65] to assess fast bowling biomechanics was judged to increase risk of bias, as too few trials may not appropriately represent long-term technique [75], and individual movement patterns and movement variability associated with fast bowling technique fluctuate within bowling sessions [76]. Increased bias was judged when MRI assessments were scheduled at a time to allow bowlers further recovery [64], and when injuries pooled for analysis were sustained prior to and following biomechanical testing [60], as the assumption of similar bowling technique before and after injury is tenuous.

The dichotomising of continuous variables into discrete categories using arbitrarily chosen or data driven thresholds [71] was judged to increase risk of bias [42–44, 46, 47, 50, 54, 55, 58–60, 65]; as this practice discards information, reduces statistical power, and is biologically implausible [77] through its assumption that all participants within a category possess equal risk of injury [78]. Studies not referencing the reliability of measurements were adjudged to possess increased bias risk [42, 43, 53–56, 59–61, 65], and whilst two studies understandably employed a multicentre approach for MRI [52, 64], inter-rater reliability for BMO detection in one of these [52] was reported as “moderate” in subsequent research [79]. Studies investigating associations between QL asymmetry and injury that could access images across limited vertebral levels [44, 46, 47] were deemed prone to increased bias risk, as was a study that reported bowling technique biomechanical variables for only 68% of recruited participants [59].

The highlighting of these potential biases can guide future researchers and approaches that include quantifying the number of fast bowling trials to provide a stable estimate of key performance and biomechanical variables, standardising protocols to improve inter-rater detection of radiological abnormalities, and accounting for previous injury in study designs and analyses. Furthermore, continuous variables should remain continuous and be modelled appropriately [71], and the validity and reliability of employed measurement tools should be established to limit misclassification bias [67].

#### Outcome measurement bias

Nine studies [44, 48, 56–58, 60, 61, 63, 65] were classified as having a moderate risk of outcome measurement bias, and seven [41–43, 49, 53–55] were rated as high risk. Increased bias was adjudged when methodologies or reliability associated with LBP and lumbar spine injury outcomes were not reported [42–44, 53–56, 65]. Potential recall bias may have caused under-reporting of injury incidence in studies relying on retrospective data sourced from participants [48, 60, 61], or from medical records [57, 58, 63]. Cross-sectional [55, 56, 62, 63], retrospective [45, 54, 57–59, 61, 64], and prospective studies with one cricket season follow up [42, 46–50, 52, 61] were deemed to exhibit increased bias due to missing, adverse, or otherwise injury outcomes that may have occurred over a prolonged period of observation [80].

Studies that employed CT to investigate LSF presence [42, 54, 55] are prone to misclassification bias [67, 81], as CT possesses reduced sensitivity in diagnosing stress reactions [81]. Research categorising injury on radiological findings alone [43, 53–56, 65] or studies including both asymptomatic bowlers with abnormal radiology and bowlers with symptoms [41, 49] were considered to display increased risk of bias. Reported dissociations between lumbar symptoms and MRI [31, 52] and CT [82] detected abnormalities support this judgement, as a proportion of fast bowlers with radiological abnormalities will not experience symptomatic injuries and missed playing time [31, 52, 82].

Multiple injury definitions within and between studies confound the relevance of investigated variables [37], and future studies should employ injury definitions that encompass symptoms, clinical signs, and imaging findings, as these may better represent injury burdens in fast bowling cohorts. The creation of a multidisciplinary consensus for LBP and lumbar spine injury diagnosis in fast bowlers may be an important step in improving the external validity of future research.

#### Confounding bias

Future studies investigating causality should carefully consider the concept of confounding bias, as injury is the result of a complex interplay between tissue loading and a range of modifiable and non-modifiable physiological factors including tissue specific mechanical properties and adaptations that affect tissue resilience [15, 83]. Studies implying causality that did not account for confounders [43, 45, 50, 53, 55, 58, 60–62, 65] were rated as having a high risk of confounding bias; whereas those that accounted for a limited number of confounders [48, 51, 52, 63, 64] or measured confounders and did not account for them in a multivariate analysis [42, 54, 56] were rated as moderate risk. Reportedly significant associations in these studies may have been distorted by confounders that were related to an investigated variable as well as LBP or lumbar spine injury.

Future projects should incorporate directed acyclic graphs (DAGs), as these can illustrate confounders to include and adjust for and improve the understanding of mediating effects and bias implications of confounders [84, 85]. Furthermore, the effect of investigated variables and confounders on injury risk may change over time [15, 86] due to changes in the mechanical properties of muscle, tendon, and bone tissue [83] in response to training and match loads. Whilst several included studies accounted for bowling workloads [51, 52, 64], future research should longitudinally account for multiple variables as time-varying effect-measure modifiers and/or time varying confounders [87].

#### Statistical analysis and reporting bias

Studies classified as having a high risk of statistical analysis and reporting bias were typified by inadequate reporting [42, 54, 55] and when the 95% Confidence Intervals of risk ratios for variables reported to be significant included 1.0 [49, 64]. Studies with reported associations based on *p*-values alone [43, 45, 48, 56, 57, 60, 62, 65] were rated as moderate risk, as *p*-values do not provide a good measure of evidence regarding a hypothesis or quantify the size of an effect [88]. The use of post-hoc power analyses to detect differences in bowlers with and without injury [46, 47] was judged to increase risk of bias, as it is not conceptually valid to interpret power pertaining to observed study results [89], as this should be included in study rationale and design prior to conduct [90].

Low risk (and primarily more recent) studies [41, 44, 50–53, 58, 59, 61, 63] were characterised by appropriate interpretations of associations based on effect sizes [41, 50, 53, 58, 61, 63], relative risk ratios [50], predictive values [52], and regression models [44, 51, 53, 59]. Whilst the inclusion of these measures to accompany *p*-value and Confidence Interval reporting is promising, further steps are required to produce more transparent and informative research. Approaches such as Bayesian methods, likelihood ratios, and Bayes Factors may more directly address the size and certainty of effects, or whether a hypothesis is correct [71, 88]. Furthermore, future researchers should report both relative and absolute measures of association to draw conclusions, as these may better identify minimal important differences in injury risk [91].

### Summary of evidence

Notwithstanding reported discrepancies, the credibility of extracted results in this review are potentially compromised due to 23 of the 25 included studies being assessed as having an overall high risk of bias. Regardless, discussion within this context of the summary of evidence can inform priorities and strategies for future research.

#### Age

Inconsistent associations of age and injury in adolescent populations [41, 54, 59] may be due to disparate study designs and injury outcomes of included studies; as radiological abnormalities were assessed in a cross-sectional study [54], symptomatic LBSI was examined in retrospective research [59], and a combination of these outcomes was investigated prospectively [41]. The association of younger age and LBSI in professional bowlers [51] is supported by research reporting fast bowlers with LBSI possessing mean age of 22.2 years [13], being predominantly 24 years or younger [92], and demonstrating 3.7 to 6.7 times greater likelihood of sustaining these injuries than other age groups if they are under the age of 22 [20].

These findings are consistent with longitudinal measurements of bone turnover and BMD indicating lumbar bone accrual continues beyond longitudinal growth cessation [93–95], with 23.1 to 24.9 years reported as the 95% Confidence Interval for attainment of peak BMD in males [95]. The lumbar vertebrae undergo maturation at secondary ossification centres in the vertebral body ring [96–98], and the mamillary, transverse and spinous processes [96] through cartilaginous, apophyseal and epiphyseal stages [98] that do not correlate consistently with chronological age [96–99]. Furthermore, the timing and rates of growth and maturation of lumbar musculature are variable; as Erector Spinae and Multifidus often reach maximal CSA before skeletal maturity, whereas Psoas Major and QL can continue to increase in size after skeletal maturity [100].

Previous reviews examining LBP and lumbar spine injury in fast bowlers have classified adolescent [26] and adult [16] cohorts separately, with caution advised in generalising injury associated factors from cricketers below 18 years to adults [16, 26]. Whilst disparities in spinal anatomy exist between adolescents and adults [101], future research should examine neuromuscular [102], physiological and mechanical adaptations [83] as a function of training age [83, 103] and maturation status [102], as these factors may influence resilience to fast bowling [1]. Regardless, a linear relationship between skeletal maturity and chronological age is disputable [104], and the rationale for classifying fast bowlers by chronological age should be re-considered. Whilst one included study [41] reported the non-significance of skeletal age, future studies should investigate associations of lumbar maturation metrics and injury outcomes.

#### LBP and lumbar spine injury

Irrespective of study design, the residual effects of previous injury potentially distort reported associations between investigated variables and reported outcomes of LBP and lumbar spine injury. An index LBSI in a fast bowler may be associated with recurrence at the same site, contralaterally, or at a different lumbar level [13, 105]; however, the nature of any association is undetermined, as the effects of injury on the cellular and mechanical aspects of lumbar bone are unknown. Future studies should clearly define the nature and site of previous injuries and employ statistical designs to account for their influence on investigated variables and injury incidence.

Comparing the significance of variables derived from studies that defined outcomes as varied as LBP in the absence of radiology [48, 50, 57, 58, 61, 62], radiological abnormalities in the absence of LBP [43, 53–56, 65], or a combination of LBP, abnormal radiology and missed playing time [42, 44–47, 51, 52, 59, 60, 63, 64] is problematic. Whilst the appearance of LBP has been reported to be a common finding in fast bowlers without accompanying missed playing time [9]; spondylolysis is the most common cause of LBP in young athletes [106], and LBSI should be suspected in a fast bowler presenting with LBP contralateral to their bowling arm side [12]. The significance of LBP as a surrogate for lumbar spine injury in fast bowlers is yet to be determined due to previously described attrition biases in the published literature [41–43, 45–50, 53, 60, 61, 65], and studies being cross sectional [54–56, 62, 63], retrospective [57–61, 64], or prospective with limited follow up periods [42, 46–50, 52, 61]. To better understand the relationship between LBP and lumbar spine injury in fast bowlers, there is a requirement for longitudinal studies that concurrently examine these outcomes over prolonged surveillance periods.

#### BMC/BMD

Injury causality cannot be established in cross-sectional research that reported trends for less marked asymmetry of lumbar BMC/BMD being associated with LSF history [63]. These trends are supported by lumbar vertebral BMC/BMD being reduced at 21 to 24 weeks post LSF in fast bowlers in comparison to baseline [105]; and a post-injury delay in BMC/BMD recovery may be associated with LBSI recurrences [105]. The contrasting trend for bowlers with greater contralateral side BMD who developed LBSI [41] suggests that LBSI risk may be somewhat independent of BMD. Future studies should aim to investigate additional factors that may regulate bone modelling and adaptation to mechanical loading such as vitamin D status, genetic variants associated with vitamin D and collagen pathways [107], and vertebral trabecular bone quality [108].

#### Trunk and lumbar muscle morphology and morphometry

Contrasting QL asymmetry associations may be consequential of a limited number of images available for analysis [44, 46, 47], and distinctive CSA [46, 47] and volumetric [44] assessments. The generation of volumes via muscle profile templates over multiple years [44] possibly distorted measurements, as QL CSA growth is non-uniform during adolescence [100]. Disparities may also reflect variability in the size, number, and attachments of QL fascicles between individuals [109]. Whilst increases in asymmetry have been linked to higher lumbopelvic lateral flexion loads in fast bowlers [110], finite element modelling suggests that asymmetry may reduce lumbar loads due to the geometrical proximity of QL’s line of action to the centre of spinal rotation and impacted facet joints during fast bowling postures [111].

The hypothesis of modified trunk control explaining the association between reduced non-bowling arm side Internal Oblique thickness and LBP [62] is problematic as bowling workloads were not accounted for as a confounder in this cross-sectional study. LBP-related reductions in bowling volume and intensity prior to testing may have influenced this finding, as repetitive fast bowling can preferentially hypertrophy the non-bowling arm side Internal Oblique over the course of a cricket season [112].

Since fatty infiltration within trunk and lumbar musculature has been associated with LBP [113], future research should quantify lean muscle mass. Whilst individual muscles are postulated to influence lumbar function due to spinal and fascial attachments [114], lumbopelvic function depends on coordinated activation [115] rather than specific muscles with unique architectural properties or mechanical advantages [116]. High levels of paraspinal and gluteal muscle activation have been reported around BFC and BR in injury-free fast bowlers [21], and whilst the applicability of these findings is unknown, further research is required to establish the role of trunk and lumbopelvic musculature in LBP and lumbar spine injury in fast bowlers.

#### Presence of BMO

Distinctions in associations between BMO and future lumbar spine injury may be attributed to studies reporting the significance of detected BMO [45, 52] and BMO intensity ratios [64] with *p*-values [45], predictive values [52], and risk ratios [52, 64]. Earlier symptom reporting with detected BMO and cortical breach in comparison to no cortical breach [45] may be indicative of bowlers being at a later stage of the bone stress injury continuum at study commencement [117].

Whilst excellent reliability for BMO intensity quantification has been reported [64, 79]; the inter-rater reliability of BMO detection is uncertain, as its Kappa was 0.483 (95% CI 0.368–0.580) [79] in relation to one cohort [52] and not reported in two others [45, 64]. The reporting of BMO intensity ratio ≥ 2.0 resulting in a 1.8 times greater risk of sustaining LBSI in the following 12 months should be viewed cautiously as the 95% Confidence Interval associated with its risk ratio included 1.0 (95% CI 0.6–5.5) [64].

Whilst a relative risk of 22.3 was reported for detected BMO leading to a symptomatic LBSI in adolescents, 61% of all participants with BMO detected on one or more scans did not experience a symptomatic LBSI; with 37% experiencing persistent BMO and no symptoms, and the remaining 24% experiencing BMO resolution and no symptoms [52]. The undetermined significance of BMO presents implications for its measurement in future research, and in addition to BMO detection and intensity, the quantification of lumbar intervertebral disc degeneration [118], vertebral morphometry [119], trabecular bone quality [108], paraspinal muscle morphometry [120], facet orientation [121], and facet degeneration [122] may be prudent, as these variables potentially influence relationships between quantified BMO and symptoms.

#### Biomechanics of fast bowling

Reported associations from research relating to front-on shoulder alignment at BFC [42, 54], SCR [42, 54, 56, 65] and a mixed technique [43, 54–56, 65] were replicated in only one [60] of six subsequent studies [49, 53, 58–61]. Likewise, disparities exist between the reported significance of trunk and lumbar lateral flexion [49, 53, 58, 59, 61], flexion/extension [49, 53, 58, 61], and rotation [49, 53, 58, 61], as well as hip [42, 49, 53, 60] and knee angles [42, 49, 53, 56, 60, 65] during FFC. The lack of reproducibility in biomechanical research is concerning since the modification of shoulder alignment, SCR, a mixed technique, trunk lateral flexion, and lower limb kinematics are emphasised in contemporary injury prevention and coaching programs [123, 124].

The non-consensus of predisposing or predictive biomechanical variables is understandable as these were gathered from research with disparate cohorts and study designs. Biomechanical research was conducted across adolescent [42, 43, 49, 54–56, 59, 61, 65] and adult [53, 57, 58, 60, 61] cohorts at club/school [42, 43, 56], high-performance/elite [53–55, 57–61, 65], and diverse [49] skill levels. The utility of biomechanical factors from cross-sectional [54–56] and retrospective [57–61] cohorts for injury prediction and prevention is questionable, since fast bowling techniques employed by these bowlers may have been influenced by existing or previous injury, as pain may alter muscle activity and mechanical behaviours at multiple levels of the motor system [125]. Furthermore, as previous LBP or lumbar spine injury was documented in only one biomechanical study [61], the significance of biomechanical factors collected from prospectively monitored cohorts that did not account for this [42, 43, 49, 60, 65] may be similarly affected.

Precise temporal characteristics of BFC and FFC were reported in only three studies [53, 58, 61], and their limited reporting [42, 43, 49, 54–56, 59, 60, 65] may have caused variables across biomechanical research to be collected from arbitrary and inconsistent points of the fast bowling action. Formulating precise and consistently defined parameters for the measurement and reporting of these events is necessary to improve the external validity of research. The restriction of data analysis in all included biomechanical studies [42, 43, 49, 53–56, 58–61, 65] to discrete time points or joint and/or segmental maxima and minima, represents a clear limitation [126], and future research needs to assess coordinated movement patterns utilising continuous datasets over the entire fast bowling movement [127]. Moreover, assessing variability in movement may provide an improved understanding of stresses that potentially reduce or increase cumulative loads on internal structures [76, 128] of fast bowlers, and these detailed analyses can be achieved with time-series based procedures such as Statistical Parametric Mapping [126, 129].

The threshold for SCR deemed to be “excessive” was inconsistent, with this being greater than 10° [54–56], 20° [43, 65], 30° [53, 58, 60] or 40° [59]. 2-D studies [42, 43, 54, 56, 59, 65] captured kinematic data with one high-speed camera positioned laterally and another overhead, thus introducing the risk of perspective error due to the multi-planar nature of fast bowling [5]. Moreover, SCR is a 2-D description of shoulder alignment in the transverse plane [60] that does not consistently represent 3-D derived fast bowling spinal kinematics [58, 130, 131], and bowlers classified with mixed and non-mixed actions exhibited no significant differences in lower trunk extension, lateral flexion, and axial rotation percentages utilised in the fast bowling action [130]. These findings further indicate a requirement for future research to investigate alternative methods of analysis and classification of fast bowling techniques.

Whilst 3-D studies [49, 53, 58, 60] utilised numerous cameras to reconstruct a three-dimensional space, they defined the thoracic and lumbar spine as singular rigid body segments [126]. Individual thoracic and lumbar vertebrae move in an uncorrelated manner [132, 133], and the L4 and L5 posterior elements may experience dissimilar maximal stresses in response to an applied physiological load [134]. Biomechanical data collected with rigid segments should be viewed with caution [133], and future research must explore methodologies capable of assessing multi-segmental motion of the thoracic and lumbar regions [133] and the quantification of spinal curvature [126] during fast bowling.

The significance of excessive lateral flexion in relation to injury thorax [49, 58], thoracolumbar [53], lumbopelvic [49, 53], trunk [59] and lumbar [61] segments is uncertain. In studies that have reported excessive lateral flexion to be significant, its presence may have predated or be consequential of LBP history [58], and it has been associated with both symptomatic and asymptomatic bowlers [49, 53]. In described conference proceedings, fast bowlers who sustained LBSI utilised lower proportions of available lower trunk lateral flexion range and a non-significantly increased amount of lower trunk extension than non-injured counterparts [10].

Increases in lumbar extension at BFC [61] and FFC [53] deemed to elevate LBP and LBSI risk respectively were coexistent with increased anterior pelvic tilt in one study [53], and analysis of continuous datasets may illuminate causal interactions between these variables and injury. Whilst the described influences of thoracic and lumbar rotation [49, 53, 61] are conflicting, reported increases in thoracic rotation away from the direction of delivery in bowlers without LBP history [61] may inform biomechanical and physical preparation initiatives designed to reduce lumbar spine stress during fast bowling. More detailed analyses of lateral flexion, extension and rotation are required as these movements have been designated as injurious due to hypothesised stresses on posterior lumbar vertebral elements [134, 135].

The reported association of increased rear hip flexion at BFC instant with LBSI is proposed to be representative of poor pelvifemoral control [53], which is defined by the interaction of the pelvis on the femur [136]. Whilst no reliable or valid test for this entity exists, this is associated with perceptions amongst cricket coaches that rear leg kinematics are important determinants of fast bowling performance [137], and future research investigating the origins and consequences of observed kinematics at BFC may inform interventions to improve performance and reduce injury.

Although reduced front hip flexion during FFC is theorised to cause injury due to higher GRF [42, 60] and forces transmitted to the lumbar region [49]; more flexed front hip angles in conjunction with increased pelvic anterior tilt and ipsilateral drop are also proposed to be detrimental [53]. Whilst a straighter front knee during FFC [42, 60] is suggested to be a trade-off between improved bowling performance and heightened injury risk [138]; foot horizontal impulse magnitude [139], plant angle and strike position [140] during FFC may be more important determinants of knee kinematics and GRF.

The absence of consistent findings relating high GRF at FFC with injury [42, 49, 53, 54, 56, 60, 61] may reflect discrepancies of GRF in laboratory versus match settings due to shortened run-up lengths and altered foot placement strategies [30]. Attainment of match intensity bowling speeds during testing may improve the ecological validity of future research, as reduced bowling speeds may cause disproportionately large reductions in lumbar loads [141]. IMUs may contribute to the assessment of match intensity fast bowling kinetics in future studies, as they can be used in a field environment and have good measurement validity [61, 142].

GRF dissipation may supersede the influence of GRF magnitudes since reduced force attenuation during landing may increase stresses on more proximal structures [143, 144]. Investigating the force attenuating ability of the lower limbs and lumbopelvic region through eccentric strength [145], range of motion [146, 147], and stiffness [148, 149] assessments should be considered in future research. Whilst higher lumbar flexion and lateral flexion loads have been associated with injury [49], previous research has not investigated the influence of muscle forces on lumbar compressive loads [6, 49, 150], and future studies should incorporate musculoskeletal models that better simulate spinal loading [151].

As biomechanical research has been conducted in primarily male and most likely Caucasian populations, the generalisability of findings to females and other racial groups may be limited. Female fast bowlers may adopt a bowling technique where BR speed is contributed to more by whole body angular momentum and pelvis and trunk rotation about the longitudinal axis in comparison to their male colleagues [152]. Furthermore, during landings, males demonstrate greater centre of mass displacement indicating a softer landing technique than females to absorb forces experienced during initial ground impact [153]. Differences in neuromuscular control strategies adopted during fast bowling and landing may alter the influence of extrinsic and intrinsic variables on the development of LBP and lumbar spine injury in female fast bowlers, thus indicating a requirement for future research in female fast bowling populations.

#### Physical characteristics

Conclusions from included studies investigating physical characteristics [41, 42, 46–49, 54, 56, 57, 59] may inform contemporary programs for injury prevention [154, 155]. Prospective [41, 42, 46–49], cross-sectional [54, 56], and retrospective [57, 59] designs, as well as indeterminate reliability of musculoskeletal screening procedures [156] may have contributed to inconsistencies in results.

The reporting of a reduced lumbo-pelvic stability test score resulting in a 1.7 times increased risk of sustaining lower back injury should be viewed cautiously as the 95% Confidence Interval associated with its risk ratio included 1.0 (95% CI 0.78–4.10) [49]. This test is based on the Sahrmann five-level core stability test [157], which displays questionable reliability and validity; as abdominal activity does not sequentially increase during its ascending levels [158], and the ICC for test–retest reliability is moderate (*r* = 0.649) [159]. Future research should examine methodologies to assess lumbopelvic stability in upright positions evaluating dynamic lumbar spine and pelvis control in sagittal, frontal, and transverse planes of motion over the weightbearing leg [160, 161].

The assessment of pelvifemoral stability with a single leg decline squat conceptually lacks validity, as the presence of a decline alters femoral rotation and knee valgus [162], displaces the body’s centre of mass posteriorly [163], and reduces hip strength required to control knee alignment [162, 164]. Future assessments of hip control should focus on hip muscle strength, and multiplanar knee, femoral, pelvis and spine alignment in single leg stance [165]. Whilst reduced lumbar proprioception may result in increased end-range lumbar loading [166], assessing this at speeds more representative of fast bowling with simulated back and front foot landings may be valuable in future studies.

The significance of reduced lumbar extensor endurance in fast bowlers is uncertain as it was investigated in three studies [49, 57, 59] and associated with injury in only one [49]. Future research investigating the significance of lumbar extensor strength and endurance should quantify lumbar sagittal curvature and extensor muscle volume, as these factors may influence the magnitude of muscle forces required for biomechanical stability of the lumbopelvic region [167, 168].

### Limitations

The heterogeneity of investigated variables did not enable a meta-analysis to be performed, and yet to be identified variables may be associated with LBP and lumbar spine injury. Whilst the level of agreement of risk assessments for individual QUIPS bias domains was high, final judgements were subjective to some degree. It is possible that relevant articles were not identified during the search process, and studies published in languages other than English may have been overlooked. Positive publication bias was a likely factor in the non-retrieval of studies, and the exclusion of grey literature and conference proceedings may have contributed to this.

## Conclusion

This review has identified inconsistencies in findings from studies investigating associations between intrinsic variables and LBP and lumbar spine injury. These discrepancies may be related to differences in study design, injury definitions, participant characteristics, measurement parameters, and statistical analyses. LBP and lumbar spine injury occurrence in fast bowlers remain high, and this may be due to an absence of low bias studies that have informed recommendations for their prevention. Careful study design, precise measurement, appropriate statistical analysis, and clearly defined measurement and injury outcomes represent important strategies for minimising bias and improving the representativeness of findings. Future research should prioritise analysis of continuous datasets, models that better represent lumbar kinematics and kinetics during fast bowling, and improved quantification of previous injury, lumbar anatomical features and lumbar maturation.

### Supplementary Information

**Additional file 1.** Presents a detailed search strategy for each database.

**Additional file 2.** Presents detailed and defined criteria for the rating of studies using the Quality in Prognostic Studies (QUIPS) tool.

**Additional file 3.** Presents detailed information regarding individual Risk of Bias assessments for each of the Quality in Prognostic Studies (QUIPS) domains of each included study.

**Additional file 4.** Presents definitions for intrinsic variables that have been associated with low back pain and lumbar spine injury in fast bowlers.

## Data Availability

All data and material reported in this systematic review is from peer-reviewed publications. All extracted data is included in this published article [and its supplementary information files].

## Abbreviations

2-D: Two-dimensional
3-D: Three- dimensional
BFC: Back foot contact
BMC: Bone mineral content
BMD: Bone mineral density
BMI: Body mass index
BMO: Bone marrow oedema
BR: Ball release
CHARMS: Checklist for critical Appraisal and data extraction for systematic Reviews of prediction Modelling Studies
CI: Confidence Interval
CL: Contralateral
CSA: Cross sectional area
CT: Computed Tomography
DAGs: Directed acyclic graphs
DEXA: Dual Energy X-ray Absorptiometry
EO: External Oblique
F/E: Flexion/extension
FFC: Front foot contact
GRF: Ground reaction forces
HSS: Hip-shoulder separation
IMUs: Inertial measurement units
IO: Internal Oblique
LBP: Low Back Pain
LBSI: Lumbar bone stress injury
LRE: Lumbar Reposition Error
LSF: Lumbar stress fracture
MF: Multifidus
MRI: Magnetic Resonance Imaging
QL: Quadratus Lumborum
QUIPS: Quality in Prognostic Studies
ROC: Receiver operating characteristic
RR: Risk ratio
SCR: Shoulder counter rotation
SEBT: Star Excursion Balance Test
SPECT: Single Photon Emission Computerised Tomography
US: Ultrasound
