# Supplementary material for: Intrinsic variables associated with low back pain and lumbar spine injury in fast bowlers in cricket: a systematic review
Source: BMC Sports Sci Med Rehabil. 2023 Sep 20;15:114. doi: 10.1186/s13102-023-00732-1 (PMC10512628; doi:10.1186/s13102-023-00732-1)
Supplement: Supplementary file 1 — Additional file 1. Presents a detailed search strategy for each database. [file 13102_2023_732_MOESM1_ESM.docx]

**Additional File 1 Database Search Strategy**

| Search | OVID Medline/EMBASE |
| --- | --- |
| # 1 | exp Cricket / |
| # 2 | exp * Fast Bowling / |
| # 3 | exp * Fast Bowler / |
| # 4 | 1 OR 2 OR 3 |
| # 5 | Risk Factor*/ |
| # 6 | Risk / |
| # 7 | Factor* / |
| # 8 | Variable / |
| # 9 | Intrinsic / |
| # 10 | Age / |
| # 11 | Adolescent / |
| # 12 | Young / |
| # 13 | Adult / |
| # 14 | Technique / |
| # 15 | Biomechanic* / |
| # 16 | Kinematic* / |
| # 17 | Kinetic* / |
| # 18 | Strength / |
| # 19 | Flexibility / |
| # 20 | Range of motion / |
| # 21 | Muscle / |
| # 22 | exp * Asymmetry / |
| # 23 | exp *Cross Sectional Area / |
| # 24 | exp * Volume / |
| # 25 | 5 OR 6 OR 7 OR 8 OR 9 OR 10 OR 11 OR 12 OR 13 OR 14 OR 15 OR 16 OR 17 OR 18 OR 19 20 OR 21 OR 22 OR 23 OR 24 |
| # 26 | Pain / |
| # 27 | Injury / |
| # 28 | Fracture / |
| # 29 | * Stress / |
| # 30 | exp Stress Fracture / |
| # 31 | exp Stress Reaction / |
| # 32 | Reaction / |
| # 33 | Pars Interarticularis / |
| # 34 | Pars / |
| # 35 | Pedicle / |
| # 36 | exp * Spondylolysis / |
| # 37 | exp * Spondylolisthesis / |
| # 38 | Bone / |
| # 39 | exp Oedema / |
| # 40 | exp Edema / |
| # 41 | 26 OR 27 OR 28 29 OR 30 OR 31 OR 32 OR 33 OR 34 OR 35 OR 36 OR 37 OR 38 OR 39 40 |
| # 42 | Low Back / |
| # 43 | Back / |
| # 44 | Lumbar / |
| # 45 | Lumbar spine / |
| # 46 | 42 OR 43 OR 44 OR 45 |
| # 47 | 4 AND 25 AND 41 AND 46 |

KEY:

/ - denotes use of Medical Subject Headings (MeSH) in OVID Medline and EMTREE in EMBASE

exp - subject heading has been exploded to include narrower more specific terms

* prior to search term - subject heading has been focussed

* following search term - Truncation (search term starting with the letters preceding the asterisk)

SCOPUS search (limited to Medicine and Health Professions)

( "Cricket" OR "Fast Bowling" OR "Fast Bowler" ) AND ( "Risk Factor*" OR "Risk" OR "Factor*" OR "Variable" OR "Intrinsic" OR "Age" OR "Adolescent" OR "Young" OR "Adult" OR "Technique" OR "Biomechanic*" OR "Kinematic*" OR "Kinetic*" OR "Strength" OR "Flexibility" OR "Range of motion" OR "Muscle" OR "Asymmetry" OR "Cross Sectional Area" OR "Volume" ) AND ( "Pain" OR "Injury" OR "Fracture" OR "Stress" OR "Stress Fracture" OR "Stress Reaction" OR "Reaction" OR "Pars interarticularis" OR "Pars" OR "Pedicle" OR "Spondylolysis" OR "Spondylolisthesis" OR "Bone" OR "Oedema" OR "Edema" ) AND ( "Low Back" OR "Back" OR "Lumbar" OR "Lumbar Spine" ) AND ( LIMIT-TO ( SUBJAREA,"MEDI" ) OR LIMIT-TO ( SUBJAREA,"HEAL" ) )

SPORTDiscus and CINAHL Search retrieval steps

|  | ( "Cricket" OR "Fast Bowling" OR "Fast Bowler" ) |
| --- | --- |
| AND | ( "Risk Factor*" OR "Risk" OR "Factor*" OR "Variable" OR "Intrinsic" OR "Age" OR "Adolescent" OR "Young" OR "Adult" OR "Technique" OR "Biomechanic*" OR "Kinematic*" OR "Kinetic*" OR "Strength" OR "Flexibility" OR "Range of motion" OR "Muscle" OR "Asymmetry" OR "Cross Sectional Area" OR "Volume" ) |
| AND | ( "Pain" OR "Injury" OR "Fracture" OR "Stress" OR "Stress Fracture" OR "Stress Reaction" OR "Reaction" OR "Pars interarticularis" OR "Pars" OR "Pedicle" OR "Spondylolysis" OR "Spondylolisthesis" OR "Bone" OR "Oedema" OR "Edema" ) |
| AND | ( "Low Back" OR "Back" OR "Lumbar" OR "Lumbar Spine" ) |

Web of Science Search

(((TS=(( "Cricket" OR "Fast Bowling" OR "Fast Bowler" ))) AND TS=(( "Risk Factor*" OR "Risk" OR "Factor*" OR "Variable" OR "Intrinsic" OR "Age" OR "Adolescent" OR "Young" OR "Adult" OR "Technique" OR "Biomechanic*" OR "Kinematic*" OR "Kinetic*" OR "Strength" OR "Flexibility" OR "Range of motion" OR "Muscle" OR "Asymmetry" OR "Cross Sectional Area" OR "Volume" ))) AND TS=(( "Pain" OR "Injury" OR "Fracture" OR "Stress" OR "Stress Fracture" OR "Stress Reaction" OR "Reaction" OR "Pars interarticularis" OR "Pars" OR "Pedicle" OR "Spondylolysis" OR "Spondylolisthesis" OR "Bone" OR "Oedema" OR "Edema" ))) AND TS=(( "Low Back" OR "Back" OR "Lumbar" OR "Lumbar Spine" ))
