# Supplementary material for: Intrinsic variables associated with low back pain and lumbar spine injury in fast bowlers in cricket: a systematic review
Source: BMC Sports Sci Med Rehabil. 2023 Sep 20;15:114. doi: 10.1186/s13102-023-00732-1 (PMC10512628; doi:10.1186/s13102-023-00732-1)
Supplement: Supplementary file 2 — Additional file 2. Presents detailed and defined criteria for the rating of studies using the Quality in Prognostic Studies (QUIPS) tool. [file 13102_2023_732_MOESM2_ESM.docx]

**Additional file 2 Defined criteria for rating of included studies with the Quality in Prognostic Studies (QUIPS) tool**

1. Study Participation

| *Prompting items* | *Criteria for yes (yes, no, unclear, not reported)* |
| --- | --- |
|  | Goal: To judge the risk of selection bias (likelihood that the relationship between prognostic factors and lumbar spine injury is different for participants and eligible non-participants). |
| Optimal study or characteristics of unbiased study | QUIPS criterion: the study sample adequately represents the population of interest. |
| a) Adequate participation in the study by eligible persons. | The participants should be representative of the cricket fast bowling population. The involvement of participants belonging to a single local organisation such as a club, team, academy, school, etc. has low generalisability and low representativeness. Teams and single organisations differ in training methods and strategies, competitive level of matches, sociodemographic characteristics, as well as injury recording personnel, procedures, and definitions. The interpretation of representativeness is also based on the context in the study introduction specifying the population of interest. |
| b) Description of the source population or population of interest | Studies should provide basic information about the population of interest such as the estimation of the total number and playing/skill level of participants. This is necessary to understand the representativeness of the sample and/or the context of applicability of the results. Groups of fast bowlers made up of overwhelmingly professional or adult populations may disproportionately represent survivors, as lumbar stress injuries appear to overly affect younger populations and many injured young bowlers may not return to a preinjury level of fast bowling and have an opportunity to progress to elite high performance and professional squads. |
| c) Description of the baseline study sample | As a minimum, studies should report age range and mean, gender, anthropometric characteristics and whether all participants were of a fast bowler skill set. More comprehensive descriptions would include playing experience, competition level, history of low back pain, injury history, presence of low back pain or injury, or other injury at study commencement. Accurately reporting time zero and clarifying the target population for generalisability are important steps in avoiding survivor bias. Studies with the potential to include participants with and without the outcome measurement at study commencement are at increased risk of bias. Studies should report if a control group was employed, and if so, if this was age matched, matched in numbers, and subject to equivalent examination and exposure. Elite performers or those in high performance squads may have access to higher quality monitoring and rehabilitation than fast bowlers in community-based programs so these bowlers may not be representative of the general fast bowling population. These considerations are applicable when examining cross sectional, retrospective and prospective study designs within high performance and professional cohorts. |
| d) Adequate description of the sampling frame and recruitment | Studies should report whether recruitment was on individual and/or team/organisation voluntary basis. The sampling frame and recruitment method of fast bowlers (newspaper or internet advertisement, telephone interviews, questionnaires, nomination by clubs or coaches, captured from database) should be adequately described, including methods to identify the sample sufficient to limit potential bias including the number and types of recruitment used. Studies should report whether recruitment of participants occurred simultaneously or at different times. If recruitment was through a process of interviews or randomisation, the response rate should be reported to help assess level of response and whether this is likely represents a sample that is applicable to other groups. In prospective studies that require recruitment through recommendation, searching of databases, social media, internet advertisement or telephone interviews, potential survivor bias may exist as injured players will be less likely to volunteer or be included in these studies, hence reducing the representativeness of these studies. Cross sectional studies and retrospective cohort study designs are also subject to survivor bias in that those fast bowlers who develop lumbar spine injuries may have stopped bowling and not be available to be included in these study designs, and those still bowling may demonstrate a ‘survivor bias’, which may lead to the underestimation of risk. |
| e) Adequate description of the period and place of recruitment | Studies should specify the period of recruitment, when they were conducted, and their duration. Reporting of time of recruitment or time of testing (preseason, during season or after season) is important. Timing of testing during the season potentially alters levels of recruitment and profile of risk factors based on assessment findings derived from testing. Additionally, the place of recruitment (setting and geographic location) should be adequately described. |
| f) Adequate description of the inclusion and exclusion criteria | Studies should report inclusion and/or exclusion criteria to clarify and justify whether there were fast bowlers excluded at study commencement, reasons for exclusion or whether fast bowlers were included at a later stage of the study. For prospective studies this should include explicit diagnostic criteria or “zero time” description that objectively decide which individuals are included or excluded from a study. For retrospective or case control studies, there should be definitions of discrete groups with and without current low back pain, history of low back pain or radiological findings. Both types of studies ideally should include physician diagnosis or explicit diagnostic codes. |
| Summary of QUIPS criteria for Study Participation | The study participation domain addresses whether the study sample is representative of the fast-bowling population on key characteristics sufficient to limit potential bias of the observed relationship between prognostic factors and outcome of lumbar spine injury. High risk: The relationship between prognostic factors and outcome is very likely to be different for participants and eligible nonparticipants. Moderate risk: The relationship between prognostic factors and outcome may be different for participants and eligible nonparticipants. Low risk: The relationship between prognostic factors and outcome is unlikely to be different for participants and eligible nonparticipants. |

2. Study Attrition

| *Prompting items* | *Criteria for yes (yes, no, unclear, not reported)* |
| --- | --- |
|  | Goal: To judge the risk of attrition bias (likelihood that the relationship between prognostic factors and outcome of lumbar spine injury is different for completing and non-completing participants). |
| Optimal study or characteristics of unbiased study | The study data available (i.e., participants not lost to follow-up) should adequately represent the study sample. |
| a) Adequate response rate for study participants | In prospective studies, has the study completion rate and/or attrition rate been reported? |
| b) Description of attempts to collect information on participants who dropped out | Has this information been included in the methods section? Have there been descriptions of attempts to collect information on participants who dropped out? |
| c) Reasons for loss to follow-up are provided | Study reports information on why participants were lost during the study so that the reasons for loss of participants can be evaluated. This would specifically refer to whether participants were lost due to random causes or because they may have been injured. In prospective studies, if there is no reporting of attrition bias, or if there is no reporting as to reasons why participants who commenced the study did not complete a study, the representativeness of the sample being measured may be reduced. Survivor bias can occur in prospective studies if participants’ willingness to remain in a longitudinal study depends on their health and injury status, as participants who are most impaired or injured are more likely to be lost to follow-up, resulting in a final study sample that has excluded the poorest faring members of the cohort. |
| d) Adequate description of participants lost to follow-up | Participants lost to follow-up are adequately described for key characteristics (age, sex, injury status, history of low back pain, radiology findings). |
| e) There are no important differences between participants who completed the study and those who did not | Does any information exist that describes whether important differences exist in key characteristics (age, sex, injury) and outcomes between participants who completed the study and those who did not? |
| Summary of QUIPS criteria for Study Attrition | The study attrition domain addresses whether participants completing the study (i.e., with follow-up data) represent the baseline sample. High risk: The relationship between prognostic factors and outcome is very likely to be different for completing and non-completing participants and/or if there is no reporting of attrition rate in study. Moderate risk: The relationship between prognostic factors and outcome may be different for completing and non-completing participants; Low risk: The relationship between prognostic factors and outcome is unlikely to be different for completing and non-completing participants |

3. Prognostic Factor Measurement

| *Prompting items* | *Criteria for yes (yes, no, unclear, not reported)* |
| --- | --- |
|  | Goal: To judge the risk of measurement bias related to how prognostic factors were measured (differential measurement of prognostic factors related to the level of outcome of lumbar spine injury). |
| Optimal study or characteristics of unbiased study | The prognostic factor is measured in a similar way for all participants. |
| a) A clear definition or description of the prognostic factor is provided | A clear definition or description of the prognostic factors being investigated are provided with clear specification of the method of measurement. |
| b) Method of prognostic factor measurement is adequately valid and reliable | Methods of prognostic factor measurement are adequately valid and reliable to limit misclassification bias. If injury or radiological features were sustained prior to prognostic factor measurement, then it is possible that prior injury may have caused bowlers to present with assessment findings during fast bowling technique analysis or musculoskeletal screening as a result of injury. The use of a test or procedure because it has been used previously or has no alternative is not considered support enough to its validity as a metric or prognostic factor measure. Studies should present evidence or reasonable plausibility to validate the use of a specific prognostic factor and the metric that is employed to measure that factor. This may include relevant external sources regarding the validity and reliability of testing and measurement of prognostic factors. This may also relate to characteristics such as blind measurement and limited reliance on recall if questionnaires are used. The study should clearly provide information (so that it is replicable) on how the metric/variable was constructed. The analysis of the kinematics and kinetics of fast bowling based on a single or a very small number of trials is likely to lead to inaccuracies in the measurement of prognostic factors related to this analysis. The measurement of kinematics of the fast-bowling action with the use of two-dimensional (2-D) motion analysis is subject to issues with reliability, particularly when different members of a cohort are assessed by multiple observers. The validity of 2-D motion analysis for fast bowling is questionable, as the kinematics of fast bowling are multi-planar and 2-D motion analysis is subject to perspective error. The measurement of bone marrow oedema and pathology with the use of MRI in multicentre studies is prone to error as inter-rater reliability for the detection of these variables is less than optimal. Tests would only be considered valid and reliable if there is literature supporting these assumptions. |
| c) Continuous variables are reported, or appropriate cut points are used | Studies discretizing continuous variables should report not only how the cut points or cut offs were calculated but also why a procedure was used and the effect of the discretization in relation to the number of events in each category. |
| d) The method and setting of measurement of prognostic factors is the same for all study participants | The devices and calculations used to measure prognostic factors are the same for all participants. Combinations of measures derived from different systems (e.g., 2-D vs three-dimensional motion analysis of biomechanical factors associated with fast bowling technique) for assessment across a single cohort is considered inadequate 1) without evidence supporting the aggregation coming from studies on the same systems used in the prognostic studies or 2) without providing sufficient data in the method sections to support the aggregation. If prognostic factors are not measured over a similar time span for all participants, then this is also considered to be a source of bias. |
| e) Adequate proportion of the study sample has complete data for the prognostic factor | An adequate proportion of the study sample (more than 80%) had prognostic factor variables available for analysis. A study should present the proportion of missing data. |
| f) Appropriate methods of imputation are used for missing prognostic factor data | Studies using listwise deletion should present evidence or support that data were missed completely at random, and sensitivity analysis to show the effect of imputation. Studies using multiple imputations (preferred method) should show the data were missed at random, and sensitivity analysis to show the effect of imputation. Single imputation is considered inappropriate and justification of the single imputation method among those available indicated. However, the rating is increased when at least the effect of single imputation is reported in a sensitivity analysis to show the effect of imputation. |
| Summary of QUIPS criteria for Prognostic Factor Measurement | The prognostic factor measurement domain addresses adequacy of measurement of prognostic factors of interest toward non-differential measurement related to lumbar spine injury. High risk: The measurement of prognostic factors is very likely to be different for different levels of the outcome of interest. Moderate risk: The measurement of prognostic factors may be different for different levels of the outcome of interest. Low risk: The measurement of the prognostic factors is unlikely to be different for different levels of the outcome of interest. |

4. Outcome Measurement

| *Prompting items* | *Criteria for yes (yes, no, unclear, not reported)* |
| --- | --- |
|  | Goal: To judge the risk of bias related to the measurement of outcome of lumbar spine injury. |
| Optimal study or characteristics of unbiased study | The outcome of interest is measured in a similar way for all participants. |
| a) A clear definition of the outcome is provided | A clear definition of lumbar spine injury according to standardised methods (which could relate to injury history/reported pain/clinically diagnosed pathology/radiology findings) is provided, including duration of follow-up and level and extent of the outcome construct. If the definition is modified or self-developed, the study justifies the changes and support the validity of the new or modified definition. Studies should also report why a particular definition was used as an outcome to avoid that the choice can be interpreted as a biased solution to overcome the problem of an insufficient number of outcomes for statistical analysis with alternative definitions. Studies should also demonstrate the conceptual relation between the outcome and the specific prognostic factor unless the appropriateness of a generic outcome (e.g., injured/not injured vs. specific type) is supported. |
| b) Method of outcome measurement is adequately valid and reliable | The definition of lumbar spine injury employed in a particular study is adequately valid and reliable to limit misclassification bias. This may include relevant outside sources of information on measurement properties and characteristics such as blind measurement and confirmation of outcome with valid and reliable tests. The study should report who collected outcome data and the method of collection. Reference or information to support the validity (e.g., accuracy) and reliability of the procedure should be provided. |
| c) The method and setting of outcome measurement is the same for all study participants | The method, setting and definition of the outcome measurement of lumbar spine injury for the concerned study is the same for all participants in that study. |
| Summary of QUIPS criteria for Outcome Measurement | The outcome measurement domain addresses the adequacy of lumbar spine injury outcome measurements toward non-differential measurement related to prognostic factors. High risk: The measurement of the outcome is very likely to be different related to the baseline level of prognostic factors, Moderate risk: The measurement of the outcome may be different related to the baseline level of prognostic factors, Low risk: The measurement of the outcome is unlikely to be different related to the baseline level of prognostic factors. |

5. Study Confounding

| *Prompting items* | *Criteria for yes (yes, no, unclear, not reported)* |
| --- | --- |
|  | Goal: To judge the risk of bias due to confounding (i.e., the effect of a prognostic factor is distorted by another factor that is related to the prognostic factor and the outcome) |
| Optimal study or characteristics of unbiased study | Important potential confounding factors are appropriately accounted for |
| a) All important confounders are measured | The study has considered and measured relevant confounders. In this context confounders are considered as a bias category (mixing of extraneous effects distorting the real effect of exposure that includes true confounders but also colliders, mediators, and effect modifiers). These could include but not be limited to a reasonably comprehensive set of variables/prognostic factors potentially related to lumbar spine injury in fast bowlers that would include but not be limited to the biomechanics of the fast-bowling action, physical factors such as strength and range of motion, individual demographics (age, gender), and bowling workloads. Confounders that should also be taken into consideration include those attributable to professional cricket and high-performance populations as opposed to those resulting from preprofessional (or community level) participation, and this could include variables such as increased workloads, differing schedules, differences in injury prevention and management, standard of post injury rehabilitation, differences in weight, age, bowling speeds, strength and conditioning programs, and levels of monitoring. When reference to important potential confounders is made in studies, there is an expectation that these variables were measured, or at least an explanation as to why these were not measured. Within this bias category, the manner in which a variable may influence the associations between the prognostic factor and the outcome should be explained (mediation, moderation, confounder, etc.), and this explanation should be reasonable/plausible. If the inference of the study is causal in the reporting of the relationship between investigated variables and lumbar spine injury outcomes, then the absence of measurement of potential confounders would relate to increased bias. |
| b) Clear definitions of the important confounders measured are provided | Study should report clearly in the text the operational definition of identified confounders. |
| c) Measurement of all important confounders is adequately valid and reliable | This information should be reported clearly in the text of the methodology of the study. Measurement may include relevant outside sources of information on measurement properties, also characteristics, such as blind measurement and limited reliance on recall). |
| D) The method and setting of confounding measurement are the same for all study participants | This information should be reported clearly in the text of the methodology of the study. |
| e) Appropriate methods are used if imputation is used for missing confounder data | A study using listwise deletion should present evidence or support that data were missed completely at random, and sensitivity analysis to show the effect of imputation. A study using multiple imputations (preferred method) should show the data were missed at random, and sensitivity analysis to show the effect of imputation. |
| f) Important potential confounders are accounted for in the study design | If important potential confounders are accounted for in the study design (e.g., matching for key variables, stratification, or initial assembly of comparable groups). These should be controlled in the design or in the analysis. |
| g) Important potential confounders are accounted for in the analysis | Important potential prognostic factors are accounted for in the analysis (i.e., appropriate adjustment), and these domains of interest include but are not limited to previous injury, anthropometric variables, the biomechanics of the fast-bowling action, physical factors such as strength and range of motion, individual demographics (age, gender), and bowling workloads. When suggesting variables as mediators, attempts to support the role of the variable as mediator should be provided. Also addressing (support) in a narrative way the mediator effect was evaluated positively. Any suggested confounder, effect modifier or moderator, collider etc. should be considered in the analysis. If the inference of the study is causal in the reporting of the relationship between investigated variables and lumbar spine injury outcomes, then the absence of potential confounders in the analysis of results would relate to increased bias. |
| Summary of QUIPS criteria for Study Confounding | The study confounding domain addresses potential confounding, or distortion of the relationship between investigated prognostic factors and lumbar spine injury outcomes by another factor. High risk: The observed effect of the prognostic factor on the outcome is very likely to be distorted by another factor related to prognostic factor and outcome, Moderate risk: The observed effect of the prognostic factor on outcome may be distorted by another factor related to prognostic factor and outcome, Low risk: The observed effect of the prognostic factor on outcome is unlikely to be distorted by another factor related to prognostic factor and outcome. |

6. Statistical Analysis and Reporting

| *Prompting items* | *Criteria for yes (yes, no, unclear, not reported)* |
| --- | --- |
|  | Goal: To judge the risk of bias related to the statistical analysis and presentation of results |
| Optimal study or characteristics of unbiased study | The statistical analysis is appropriate, and all primary outcomes are reported |
| a) Sufficient presentation of data to assess the adequacy of the analytic strategy | There is sufficient presentation of data to assess the adequacy of the analytic strategy. The study should contain sufficient information and details to understand the statistical methods used and the calculations done, and to allow replication. |
| b) The strategy for model building is appropriate and is based on a conceptual framework or model | Studies should use the appropriate model in relation to the goal of the study. If findings were interpreted as causal associations, the model should be based on a conceptual framework or model, and any causal assumptions should be reported. Although backward or forward selection are not considered optimal, these are acceptable selection methods for explorative studies providing that results are discussed in terms of associations, and not as causal links. Checking for and considering collinearity is required. Selecting variables significant in univariate analysis before multivariate analysis is conducted is considered inappropriate. Studies should justify the sample (power analysis) and support it is adequate for the analysis. Post hoc power analysis is inappropriate and cannot be used to justify the sample used. Studies should report information for understanding whether there is a risk of sparse data bias (e.g., event for each category or distribution of the outcome in relation to the prognostic factor). Injury recurrence should be considered. |
| c) The selected statistical model is adequate for the design of the study | Studies should report results showing the association of prognostic factors and lumbar spine injury (which could relate to injury history/reported pain/clinically diagnosed pathology/radiology findings), including information about statistical significance. Continuous variables are reported or (i.e., not data dependent) cut-off points are used. Increased risks of bias for statistical analysis are adjudged in studies that do not include careful interpretation of statistical test requirements in methodologies and reporting that refer to examining the sizes of effect estimates and confidence limits, the values of relative risk and odds ratios, and precise P values. Additionally, lower risk studies are characterised using multivariate analysis, and negative and positive predictive values. |
| d) There is no selective reporting of results | All variables (outcomes and prognostic factors) that are described in the methods section should be included in the results section with words or in numbers (tables, figures). Uncertainty should be reported for all measures of associations and considered in the interpretation. Similarly in the discussion, all results should be discussed without focusing on the significant ones and acknowledging inconsistencies and “unexpected predictor-outcome associations” that may suggest the model in invalid and poor generalizability. As a minimum, if there was no explanation supporting the relevance, validity of plausibility of a specific metric for a specific category of injuries, the details about injury numbers and types for each analysis and category (if a continuous predictor is discretized) should be reported. |
| Summary of QUIPS criteria for Statistical Analysis and Reporting | The statistical analysis and reporting domain addresses the appropriateness of the study’s statistical analysis and completeness of reporting. High risk: The reported results are very likely to be spurious or biased related to analysis or reporting. Moderate risk: The reported results may be spurious or biased related to analysis or reporting, Low risk: The reported results are unlikely to be spurious or biased related to analysis or reporting |
