# Supplementary material for: Intrinsic variables associated with low back pain and lumbar spine injury in fast bowlers in cricket: a systematic review
Source: BMC Sports Sci Med Rehabil. 2023 Sep 20;15:114. doi: 10.1186/s13102-023-00732-1 (PMC10512628; doi:10.1186/s13102-023-00732-1)
Supplement: Supplementary file 3 — Additional file 3. Presents detailed information regarding individual Risk of Bias assessments for each of the Quality in Prognostic Studies (QUIPS) domains of each included study. [file 13102_2023_732_MOESM3_ESM.docx]

| **Additional File 3 Detailed Quality in Prognostic Studies (QUIPS) assessment for evaluating risk of bias in each study** | | |
| --- | --- | --- |
| **Study ID: Foster et al. 1989 [42]** | | |
| **Domain** | **Risk of bias** | **Support for judgement** |
| **Study participation** | High | 82 potentially high-performance fast bowlers aged 15 to 22 from an unspecified number of club and/or school teams. Recruitment method was nomination by coaches. Injury history not specified. |
| **Study attrition** | Moderate | Prospective study with duration of one cricket season with no reporting of any attrition rates. |
| **Prognostic factor measurement** | High | A single bowling trial from three deliveries used for technique analysis. Discretization of fast bowling technique analysis and no rationale for cut off points described. No reference to reliability of risk factor measurements. |
| **Outcome measurement** | High | Clear injury definition with follow up of one cricket season. Computed Tomography (CT) valid to assess Lumbar Bone Stress Injury (LBSI) involving a cortical breach but not valid for lumbar bone stress reactions. Methodology and reliability associated with radiology reporting not referred to. |
| **Study confounding** | Moderate | Large range of potential confounders identified but not included in a multivariable analysis. Causation between investigated variables and lumbar injury outcome is implied. |
| **Statistical analysis and reporting** | High | Univariate analysis referred to in methodology. Results of statistical analyses not reported. |
| **Study ID: Elliott et al. 1992 [54]** | | |
| **Domain** | **Risk of bias** | **Support for judgement** |
| **Study participation** | Moderate | Sample of 20 fast bowlers (mean age 17.9 ± 1.6 years), who were members of a state association development squad. Injury history not specified. |
| **Study attrition** | Low | Retrospective cohort study design. |
| **Prognostic factor measurement** | High | A single bowling trial from two deliveries used for technique analysis. Back and front foot kinetic data recorded from separate trials. Discretization of fast bowling technique data. No reference to reliability of risk factor measurements. |
| **Outcome measurement** | High | CT valid to assess LBSI involving cortical breach but not for the diagnosis of lumbar bone stress reactions. Methodology and reliability associated with radiology reporting not referred to. |
| **Study confounding** | Moderate | Large range of potential confounders identified but not included in a multivariable analysis. Causation between investigated variables and lumbar injury outcome is implied. |
| **Statistical analysis and reporting** | High | Univariate analysis referred to in methodology. Precise p values, effect sizes or confidence intervals not reported. |
| **Study ID: Hardcastle et al. 1992 [55]** | | |
| **Domain** | **Risk of bias** | **Support for judgement** |
| **Study participation** | Moderate | Sample of 24 fast bowlers aged 16 to 18 years, selected for special training in a state team squad. Presence of low back pain (LBP) at study commencement referred to. Injury history not specified. |
| **Study attrition** | Low | Cross-sectional study. |
| **Prognostic factor measurement** | High | Methods of bowling technique measurement and assessment not reported. Discretization of fast bowling technique data. No reference to reliability of risk factor measurements. |
| **Outcome measurement** | High | Radiographs and CT scans assessed by three radiologists, but no reference to reliability of CT assessments. Magnetic Resonance Imaging (MRI) assessed independently. CT valid to assess LBSI involving cortical breach but not for the diagnosis of lumbar bone stress reactions. |
| **Study confounding** | High | No confounding variables accounted for in design, conduct and analysis. Causation between investigated variables and lumbar injury outcome is implied. |
| **Statistical analysis and reporting** | High | Methodology and results of statistical analyses are not presented. |
| **Study ID: Elliott et al. 1993 [56]** | | |
| **Domain** | **Risk of bias** | **Support for judgement** |
| **Study participation** | Moderate | 24 bowlers of mean age 13.7 years, selected from 5 schools. Injury history not specified. |
| **Study attrition** | Low | Cross-sectional study. |
| **Prognostic factor measurement** | High | A single trial digitised for technique analysis from a total of two deliveries. Discretization of fast bowling technique data. No reference to reliability of risk factor measurements. |
| **Outcome measurement** | Moderate | MRI scans assessed by one radiologist. No description or measurement of the reliability of this assessment reported. |
| **Study confounding** | Moderate | Large range of potential confounders identified but not included in a multivariable analysis. Causation between investigated variables and lumbar injury outcome is implied. |
| **Statistical analysis and reporting** | Moderate | Precise p values reported. Significance of a causal association reported with p value of 0.088. No effect sizes or confidence intervals are reported. Multivariate analysis not reported in methods or results. |
| **Study ID: Burnett et al. 1996 [43]** | | |
| **Domain** | **Risk of bias** | **Support for judgement** |
| **Study participation** | Moderate | 19 bowlers of mean age 13.6 years, selected from 5 schools. Injury history not specified. |
| **Study attrition** | High | Attrition rate not reported in this prospective study of 2.7 years duration in adolescents. |
| **Prognostic factor measurement** | High | A single trial from two bowling trials used for measurement of kinematics of fast bowling technique. Rationale for discretization of bowling technique data not reported. No reference to reliability of risk factor measurements. |
| **Outcome measurement** | High | Different MRI scanners used at start and end of study. Divergent outcome measures for different participants, as a case of pars fracture was included in the statistical analysis for this study analysing the relationship between bowling technique and thoracolumbar disc degeneration. |
| **Study confounding** | High | No confounding variables accounted for in design, conduct and analysis. Causation between investigated variables and lumbar injury outcome is implied. |
| **Statistical analysis and reporting** | Moderate | Significance level set at 0.05 and p values were reported. Effect sizes or confidence intervals not reported. Multivariate analysis not reported in methods or results. |
| **Study ID: Elliott & Khangure 2002 [65]** | | |
| **Domain** | **Risk of bias** | **Support for judgement** |
| **Study participation** | Moderate | 41 fast bowlers in two groups. Group 1 recruited from state cricket association development squad. Group 2 commenced study one year later, and method of recruitment not reported. Injury history not specified. |
| **Study attrition** | High | Attrition rate not reported in this intervention study of four years duration in adolescents. |
| **Prognostic factor measurement** | High | A single trial with the highest ball release speed from three bowling trials used for measurement of kinematics of fast bowling technique. Rationale for discretization of bowling technique data not reported. No reference to reliability of risk factor measurements. |
| **Outcome measurement** | Moderate | Each scan over the four years of the study assessed by the same blinded professional, but no description or measurement of the reliability of this assessment reported. |
| **Study confounding** | High | No confounding variables accounted for in design, conduct and analysis. Causation between investigated variables and lumbar injury outcome is implied. |
| **Statistical analysis and reporting** | Moderate | Precise p values reported. Significant main effects calculated with Tukey post hoc testing and reported. Multivariate analysis not reported in methods or results. |
| **Study ID: Portus et al. 2004 [60]** | | |
| **Domain** | **Risk of bias** | **Support for judgement** |
| **Study participation** | Moderate | Homogenous group of 42 high performance adult male fast bowlers recruited from visits over a three year period to a national sports institute. Recruitment sampling rate not specified. Potential survivor bias. |
| **Study attrition** | Moderate | Non-reporting of attrition rate in prospective portion of study. Attrition bias not applicable in retrospective portion of study. |
| **Prognostic factor measurement** | High | A single bowling trial analysed per participant. Kinematic data for different participants measured prior to injury and post injury and in some participants previous lumbar injury may have influenced fast bowling technique. Discretization used to classify fast bowling techniques in terms of hip-shoulder separation (HSS) at back foot contact (BFC), shoulder counter rotation (SCR) and knee angles during front foot contact (FFC). Sampling rate of three-dimensional (3-D) motion analysis varied between 50 and 100Hz. No reference to reliability of risk factor measurements. |
| **Outcome measurement** | Moderate | Potential recall bias associated with retrospective injury data. Prospective data recorded by medical professionals external to study group over several years. |
| **Study confounding** | High | No confounding variables accounted for in design, conduct and analysis. Causation between characteristics of fast bowling technique and lumbar injury is implied. |
| **Statistical analysis and reporting** | Moderate | Precise p values and correlation coefficients were reported. Multivariate analysis not reported in methods or results. |
| **Study ID: Engstrom et al. 2007 [44]** | | |
| **Domain** | **Risk of bias** | **Support for judgement** |
| **Study participation** | Moderate | Bowlers (n = 56) from club to national level, and a control group of swimmers (n = 20) recruited. Method of recruitment not specified. No reporting of injury history. |
| **Study attrition** | Low | Seven of a total of 76 participants did not complete this prospective study. Reasons for non-completion reported for three of the seven withdrawals. |
| **Prognostic factor measurement** | High | Whilst the method of measurement and analysis of reliability of Quadratus Lumborum (QL) asymmetry is clearly defined, muscle profile templates based on multiyear images were generated to overcome difficulties in measurement. These may have distorted reported volumes. Discretization of QL asymmetry data into quartiles. |
| **Outcome measurement** | Moderate | All MRI analyses blinded with respect to injury history and participant characteristics. Reliability of this measurement not reported. Frequency and method of gathering injury outcome data not reported. Uneven study durations in fast bowling and control groups. |
| **Study confounding** | Low | No confounding variables accounted for in design, conduct and analysis. Causation between QL asymmetry and lumbar injury is not specifically implied. |
| **Statistical analysis and reporting** | Low | Precise p values and confidence intervals reported. Univariate logistic regression, and area under the curve used for assessing the discriminatory power of the model to predict injury. |
| **Study ID: Stuelcken et al. 2008 [57]** | | |
| **Domain** | **Risk of bias** | **Support for judgement** |
| **Study participation** | High | Homogenous sample of elite female fast bowlers identified as "elite" by national team coach and recruited on this basis. Skill set and certain anthropometric characteristics reported. Potential survivor bias. |
| **Study attrition** | Low | Retrospective cohort study. |
| **Prognostic factor measurement** | Low | Assessed variables measured by two testers blinded to LBP status who underwent extensive training. Acceptable reproducibility in measurements prior to study commencement. Reliability of testing procedures employed referred to. |
| **Outcome measurement** | Moderate | Whilst self-reported pain had to be verified by records kept by team medical support staff, there was a possibility of under reporting of incidence of the outcome measure which was a career history of LBP. |
| **Study confounding** | Low | Several confounding variables were accounted for in the design and conduct, but these were not controlled for in analysis of results. Causation between lumbar lateral flexion range of motion and a career history of LBP not implied. |
| **Statistical analysis and reporting** | Moderate | P values reported with significance level of p ⩽ 0.05. Multivariate analysis not reported in methods or results. |
| **Study ID: Ranson et al. 2010 [45]** | | |
| **Domain** | **Risk of bias** | **Support for judgement** |
| **Study participation** | High | Homogenous sample of high-level fast bowlers identified by national lead fast bowling coach. |
| **Study attrition** | Moderate | High performance cohort monitored by team medical staff for 2 years with no documenting of rates of completion or drop out. |
| **Prognostic factor measurement** | Low | All bowlers underwent MRI scans in same machine. MRI scanning and reporting procedures reported to be reliable and full details reported elsewhere by same lead author [10]. |
| **Outcome measurement** | Low | MRI scans assessed by same radiologist using reliable classification systems. Questioning of participants at six week intervals reduces the potential for recall bias with injury definition relating to absence from cricket for seven or more days. |
| **Study confounding** | High | No confounding variables accounted for in design, conduct and analysis. Causation between detected MRI findings and subsequent development of a stress fracture is implied. |
| **Statistical analysis and reporting** | Moderate | Significance level set at 0.05 and p values reported. Multivariate analysis not reported in methods or results. |
| **Study ID: Stuelcken et al. 2010 [58]** | | |
| **Domain** | **Risk of bias** | **Support for judgement** |
| **Study participation** | High | Homogenous sample of elite female fast bowlers identified as "elite" by national team coach and recruited on this basis. Skill set and certain anthropometric characteristics reported. Potential survivor bias. |
| **Study attrition** | Low | Retrospective cohort study. |
| **Prognostic factor measurement** | Moderate | From a total of 20 to 25 maximum speed deliveries, four bowling trials selected for kinematic analysis. Precise definition of events and classifications in fast bowling action reported. Fast bowling technique data discretized on basis of previous studies. |
| **Outcome measurement** | Moderate | Whilst self-reported pain had to be verified by records kept by team medical support staff, there was a possibility of under reporting of incidence of the outcome measure which was a career history of LBP. |
| **Study confounding** | High | No confounding variables accounted for in design, conduct and analysis. Causation between characteristics of the fast-bowling technique and a career history of LBP implied. |
| **Statistical analysis and reporting** | Low | Independent samples t tests, effect sizes and correlation coefficients reported in methods and results. Multivariate analysis not reported in methods or results. |
| **Study ID: Kountouris et al. 2012 [46]** | | |
| **Domain** | **Risk of bias** | **Support for judgement** |
| **Study participation** | Moderate | Sample of 38 junior fast bowlers aged from 12 to 17 with no description of methods of recruitment. |
| **Study attrition** | Moderate | Non-reporting of attrition rate in this prospective study (that was part of a larger study) over one cricket season duration. |
| **Prognostic factor measurement** | High | Reference to reliability of this measurement by same author in previous study [58]. Limited number of images available for analysis and image slices included for analysis were at the L3–L4 vertebral levels. Discretization of QL cross-sectional area (CSA) magnitude of asymmetries based on percentages (0 < 10%, 10-20%, > 20%) |
| **Outcome measurement** | Low | When injury was suspected, radiological investigations were performed at radiology clinics based on the referring physician’s discretion. Participants injured during the study period were not necessarily assessed by the same sports physicians or radiologists. |
| **Study confounding** | Low | Moderate control for confounding with the inclusion of age, height, mass, and body mass index in analysis of results. Causal relationship between an absence of QL CSA and LBSI is not implied. |
| **Statistical analysis and reporting** | Moderate | Precise p values reported. Confidence intervals not reported. Multivariate analysis not reported in methods or results. Post hoc power analysis performed on results to determine study power to detect differences between injured and non-injured bowlers. |
| **Study ID: Kountouris et al. 2013 [47]** | | |
| **Domain** | **Risk of bias** | **Support for judgement** |
| **Study participation** | High | Homogenous group of 23 national and international level adult male fast bowlers. Recruitment through identification by respective selection panels. Potential survivor bias. |
| **Study attrition** | Moderate | High performance cohort monitored by team medical staff. No documenting of rates of completion in this prospective study (that was part of a larger study) over one cricket season duration. |
| **Prognostic factor measurement** | High | Reference to reliability of this measurement by same author in previous study [58]. Only 35% of all MR images were of sufficient quality to be included for analysis and no images of sufficient quality above the L2 mid vertebra level and below the L4/L5 disc level. Discretization of QL CSA magnitude of asymmetries based on percentages (0 < 10%, 10-20%, > 20%) |
| **Outcome measurement** | Low | A variety of clinical presentations may have been referred for radiology, and investigations ordered to confirm diagnosis were made at the discretion of sports medicine physicians. A range of radiology clinics and radiologists were involved in this study. |
| **Study confounding** | Low | Moderate control for confounding with the inclusion of age, height, mass, and body mass index in analysis of results. Causal relationship between an absence of QL CSA and LBSI is not implied. |
| **Statistical analysis and reporting** | Moderate | Precise p values reported. Confidence intervals not reported. Multivariate analysis not reported in methods or results. Post hoc power analysis performed on results to determine study power to detect differences between injured and non-injured bowlers. |
| **Study ID: Olivier et al. 2014 [48]** | | |
| **Domain** | **Risk of bias** | **Support for judgement** |
| **Study participation** | Moderate | 17 amateur fast bowlers participated after a process of randomisation. Potentially low response rate and no reporting of mean age may affect how representative the sample is. Description of basic characteristics of participants reported including injury history. |
| **Study attrition** | Moderate | Non-reporting of attrition rate in this prospective study in amateur cricket fast bowlers over one cricket season duration. |
| **Prognostic factor measurement** | Moderate | Lumbar reposition measurement described clearly. Reference made to reliability of electro goniometer used. Only one match pace delivery used to be representative of lumbar spine positioning in the bowling action. |
| **Outcome measurement** | Moderate | Some potential for recall bias during collection of LBP history. Low potential for bias for collection of in season data collected monthly. |
| **Study confounding** | Moderate | Some control for confounding with inclusion of previous injury in analysis of results. Causal relationship between lumbar proprioception and LBP is implied. |
| **Statistical analysis and reporting** | Moderate | Reporting of p values and 95% confidence intervals. Multivariate analysis not reported in methods or results. |
| **Study ID: Gray et al. 2016 [62]** | | |
| **Domain** | **Risk of bias** | **Support for judgement** |
| **Study participation** | Moderate | Homogenous sample of high-level adolescent fast bowlers with no description of method of recruitment. Nine bowlers in non-pain group and 16 in LBP group. Bowlers with pain may have been more likely to volunteer for this study. |
| **Study attrition** | Low | Cross-sectional study. |
| **Prognostic factor measurement** | Low | Detailed description of methods of measurement. Reliability of thickness measurements of abdominal musculature established elsewhere. Thickness used rather than CSA as not possible to visualize the entire muscles in the ultrasound (US) Image, but three thickness measures used for each muscle. |
| **Outcome measurement** | Low | Low risk of classification and recall bias with reporting of LBP history that may have caused participants to miss a match or practice session in the six weeks prior to muscle thickness assessment. |
| **Study confounding** | High | No confounding variables accounted for in design, conduct and analysis. Causation between abdominal muscle thickness and current/history of LBP implied. |
| **Statistical analysis and reporting** | Moderate | Reporting of p values. Post hoc analysis performed where appropriate. Multivariate analysis not reported in methods or results. |
| **Study ID: Bayne et al. 2016 [49]** | | |
| **Domain** | **Risk of bias** | **Support for judgement** |
| **Study participation** | Moderate | 15 of 46 pain-free participants excluded prior to study due to findings on MRI scan, potentially reducing representative of this sample. No description of method of participant recruitment. |
| **Study attrition** | High | 31 participants in study following initial MRI. Results report 25 participants, but no explanation or reasons given for missing six participants. |
| **Prognostic factor measurement** | Low | Four bowling trials, in which each bowler achieved their highest ball release speeds across 18 deliveries, selected for analysis. Reliability reported for bowling technique analysis. Reliability for musculoskeletal screening testing reported elsewhere [128]. |
| **Outcome measurement** | High | Diagnostic criteria for injury outcomes not applied uniformly for all study participants. Radiologically detected bone stress in asymptomatic participants employed as an outcome measure in addition to self-reported LBP during the course of the study. |
| **Study confounding** | Low | Wide range of confounders accounted for in design and analysis of results. Causation implied between lower back injuries and investigated variables including physical factors and characteristics of fast bowling technique. |
| **Statistical analysis and reporting** | High | Reporting of p values, Pearson correlation coefficients and 95% confidence intervals. Reporting of association of impaired lumbo-pelvic stability with injury when confidence interval crossed 1.0 (CI 0.78-410). Multivariate analysis not reported in methods or results. |
| **Study ID: Olivier et al. 2017 [50]** | | |
| **Domain** | **Risk of bias** | **Support for judgement** |
| **Study participation** | Moderate | 26 participants from 14 cricket clubs recruited through invitations and advertisements with past injury history and playing experience described. Response rate to recruitment not described. |
| **Study attrition** | Moderate | Non-reporting of attrition rate in this prospective study over an 8 month duration. |
| **Prognostic factor measurement** | Moderate | Detailed description of measurement method and reference to the intra-rater reliability of this measurement. Discretization of asymmetry data conducted to compare the injury status of bowlers with percentage difference of 10% or > to those with percentage difference of < 10% |
| **Outcome measurement** | Low | Incidence, body site, nature, and management of injury were captured monthly by one of the researchers using a self-administered questionnaire. |
| **Study confounding** | High | No confounding variables accounted for in design, conduct and analysis. Causation between reduced lumbar multifidus CSA and lower back pain implied. |
| **Statistical analysis and reporting** | Low | Reporting of p values, Cohen’s d effect sizes, 95% confidence intervals and relative risk ratios. Multivariate analysis not reported in methods or results. |
| **Study ID: Alway et al. 2019 [51]** | | |
| **Domain** | **Risk of bias** | **Support for judgement** |
| **Study participation** | Moderate | Large cohort of 368 fast bowlers prospectively monitored over seven years. Survivor bias possible as sample consisted of elite high performance fast bowlers playing in matches, thus may not be representative of broader fast bowling population. |
| **Study attrition** | Low | Whilst this was a prospective injury surveillance study that examined bowling workload and age as variables associated with lumbar spine injury, attrition bias was not applicable as data was extracted from a database at the conclusion of a six year period. |
| **Prognostic factor measurement** | Low | Age at time of injury calculated from injury date and extracted from ECB injury database used to prospectively monitor injuries. |
| **Outcome measurement** | Low | Injury outcome required symptomatic presentation and radiological evidence which resulted in a player being unavailable for match selection and was extracted from ECB injury database in this prospective study. |
| **Study confounding** | Moderate | Some control for confounding with the inclusion of bowling workloads in the analysis of results. Causal relationship between age at time of injury and development of a symptomatic lumbar stress fracture implied. |
| **Statistical analysis and reporting** | Low | Precise p values reported. Effect sizes calculated. Means, SDs and 95% confidence intervals were calculated. Multivariate analysis with binary logistic regression. |
| **Study ID: Alway et al. 2019 [63]** | | |
| **Domain** | **Risk of bias** | **Support for judgement** |
| **Study participation** | Moderate | Participants were from national senior or developmental squads. No explanation as to how fast bowlers were recruited. Potential survivor bias. Representativeness of sample to wider fast bowling population is uncertain. |
| **Study attrition** | Low | Cross-sectional study. |
| **Prognostic factor measurement** | Moderate | Measurement of prognostic factor clearly described and precision of repeatability of each analysis determined on same day testing. Bone mineral content (BMC), bone mineral density (BMD), and area of L3 vertebral body and posterior elements was measured, but this may not be representative of the same variables at the L4 and L5 levels which appear to be the most prevalent levels for LBSI in fast bowlers [31, 38]. |
| **Outcome measurement** | Moderate | Lumbar stress fracture (LSF) history potentially subject to misclassification bias due to sourcing of injury history performed retrospectively from existing records. Possible incidence of injury prior to bowler being monitored by available medical records. |
| **Study confounding** | Moderate | Some control for confounding with the inclusion of age and fat free mass as covariates to control for related changes in BMD and BMC in the analysis of results. A causal relationship between site-specific low BMD within the lumbar spine and lumbar stress fracture is implied. |
| **Statistical analysis and reporting** | Low | Univariate tests used to assess differences between groups with regards to injury and BMC/BMD. Age and fat free mass used as covariates to control for related changes in BMD and BMC. Means, SDs, effect sizes and 95% confidence intervals were calculated. |
| **Study ID: Kountouris et al. 2019 [52]** | | |
| **Domain** | **Risk of bias** | **Support for judgement** |
| **Study participation** | Moderate | Participants were Australian junior elite fast bowlers selected in squads preparing for and participating in a national carnival. Representativeness of sample to wider fast bowling population is uncertain. |
| **Study attrition** | Low | Participants prospectively monitored in a high-performance environment with use of Athlete Management System. Six MRI scans performed in an eight month period. |
| **Prognostic factor measurement** | Moderate | Both 1.5 and 3.0 Tesla MR scanners used. Study conducted over seven radiology centres. Analysis of reliability for detection of bone marrow oedema (BMO) not reported in the study, but subsequent analysis within this cohort showed an inter-rater reliability for the clinical detection of BMO was moderate (Kappa score of 0.483) [65]. |
| **Outcome measurement** | Low | Participants were prospectively monitored, and injury diagnosis confirmed by clinical examination and subsequent radiological investigations. |
| **Study confounding** | Moderate | Some accounting for potential confounding factors with bowling workloads monitored in conjunction with MRI findings. A causal relationship between BMO detected on MRI scanning and the development of a subsequent LBSI is implied. |
| **Statistical analysis and reporting** | Low | Relative risk and odds ratios of developing a symptomatic BSI in participants who had BMO on any of the MRI scans during the study calculated. Positive and negative predictive values calculated. A binary logistic model with an exchangeable correlation structure used. |
| **Study ID: Senington et al. 2020 [61]** | | |
| **Domain** | **Risk of bias** | **Support for judgement** |
| **Study participation** | High | Sample of 14 senior fast bowlers and 21 junior fast bowlers recruited through coaches from professional cricket clubs. Reporting of injury history. |
| **Study attrition** | Moderate | For retrospective analysis, attrition bias not applicable. For prospective analysis over one cricket season there was no reporting of attrition rate. |
| **Prognostic factor measurement** | Moderate | Measurement of investigated variables clearly described. Repeatability of each analysis and reliability of measuring instrument not described. The number of trials in which kinematic data was extracted from in this study was not specified in the methodology. |
| **Outcome measurement** | Moderate | Possible recall bias in the retrospective portion of the study. No clear reporting of compliance regarding the questionnaire relating to the prospective portion of the study. |
| **Study confounding** | High | No confounding variables accounted for in design, conduct and analysis. Causation between characteristics of the fast-bowling technique and low back pain implied. |
| **Statistical analysis and reporting** | Low | Univariate tests conducted to compare means of relevant data. Cohen’s d effect sizes calculated to provide an estimate of the magnitude or strength of certainty of the observed effect. Multivariate analysis not reported. Sample size calculation performed and based on previous study [49]. |
| **Study ID: Alway et al. 2021 [53]** | | |
| **Domain** | **Risk of bias** | **Support for judgement** |
| **Study participation** | Moderate | Sample of 50 elite fast bowlers enrolled in international performance pathway. Risk of survivor bias. Inclusion criteria for the uninjured group reduces the likelihood of this sample being representative of the broader fast bowling population. |
| **Study attrition** | Moderate | Participants prospectively monitored over multiple years, but no reference made to drop out or completion rates for participants. |
| **Prognostic factor measurement** | High | Whilst each participant bowled six deliveries, a single trial was employed for analysis of fast bowling biomechanical technique variables. No reference to reliability of risk factor measurements. |
| **Outcome measurement** | High | LBSI determined from radiological reports solely. Possible that both symptomatic and asymptomatic bowlers included in analysis of LBSI. The detection of LBSI with the use of radiology alone is not adequately valid as dissociation exists between radiology, symptoms, and missed playing time. Reliability of interpretation of MRI scans not reported. Unclear as to whether some bowlers possessed LBSI prior to technical analysis and whether some bowlers developed LBSI post technical analysis. |
| **Study confounding** | High | No confounding variables accounted for in design, conduct and analysis. Causation between characteristics of the fast-bowling technique and LBSI implied. |
| **Statistical analysis and reporting** | Low | Univariate analysis conducted with a significance level of 0.05. Effect sizes calculated for all variables. Significant variables with medium or greater effect sizes entered into binary regression model to predict key indicators for LBSI. |
| **Study ID: Taylor et al. 2021 [64]** | | |
| **Domain** | **Risk of bias** | **Support for judgement** |
| **Study participation** | High | Fast bowlers in screened group nominated by medical team to possibly benefit from the screening given their perceived risk of LBSI. Elite group of fast bowlers. Risk of survivor bias. |
| **Study attrition** | Low | Case series study. |
| **Prognostic factor measurement** | High | Timing of scan deliberate to allow bowlers an opportunity for recovery if indicated introduces measurement bias. Intra-rater and inter-rater reliability > 0.80 for 10% of the scans in measurement of BMO intensity ratio. |
| **Outcome measurement** | Low | LBSI in participants determined by a combination of clinical examination and radiology at the time of consultation in a high-performance cohort. |
| **Study confounding** | Moderate | Some control for confounding with the inclusion of bowling workloads as covariates to control for related changes in scan findings in the analysis of results. A causal relationship between detected BMO on MRI scans and LBSI implied. |
| **Statistical analysis and reporting** | High | Precise p values reported. Proportions and risk ratios calculated with a 95% confidence interval. Reporting of association of abnormal BMO with subsequent LBSI when confidence interval crossed 1.0 (CI 0.6-5.5 and CI 0.5-4.4). |
| **Study ID: Sims et al. 2021 [59]** | | |
| **Domain** | **Risk of bias** | **Support for judgement** |
| **Study participation** | Moderate | 222 youth fast bowlers from under 17 and 19 programs from six Australian states from 2015 to 2020 were included. Likelihood of survivor bias as sample made up totally of elite high performance fast bowlers, thus may not be representative of broader fast bowling population. |
| **Study attrition** | Low | Retrospective cohort study. |
| **Prognostic factor measurement** | High | No reliability analysis for bowling technique analysis from six separate locations. 68% of participants had bowling technique variables available for analysis. Ranges of shoulder counterrotation and trunk lateral flexion discretized into low (0-25 deg), moderate (25-40 deg) and high (> 40 deg) categories. Biomechanical and musculoskeletal data from different seasons pooled for purposes of statistical analysis. 2-D and 3-D analysis pooled to report analysis of fast bowling biomechanics and its relationship to injury. |
| **Outcome measurement** | Low | LBSI identified by a combination of clinical examination and radiology in high performance environments and prospectively with use of Athlete Monitoring System. |
| **Study confounding** | Low | Multiple confounding factors were identified and accounted for in a multivariate analysis. Causation between investigated variables and injuries implied. |
| **Statistical analysis and reporting** | Low | Significant variables (p < 0.05) and others based on previous research thought to be relevant were included into a multivariate model involving a binary logistic regression. |
| **Study ID: Keylock et al. 2022 [41]** | | |
| **Domain** | **Risk of bias** | **Support for judgement** |
| **Study participation** | Moderate | 40 adolescent male bowlers recruited from professional academies or schools and clubs with well-developed cricket programs. No description regarding method of recruitment. Details of baseline characteristics of bowlers provided. |
| **Study attrition** | High | 22 of the original cohort of 40 fast bowlers underwent MRI and Dual Energy X-ray Absorptiometry (DEXA) assessment at study conclusion. Attrition rate of 45%. Reasons given for participants who dropped out are reported. |
| **Prognostic factor measurement** | Low | DXA used to assess BMC, BMD, and skeletal maturity. A musculoskeletal assessment protocol employed prior to bowling and found to be reproducible in a preliminary reliability study (ICC ≥ 0.946). |
| **Outcome measurement** | High | Asymptomatic and symptomatic cases that presented with changes on MRI classified as LBSI cases. |
| **Study confounding** | Low | A wide range of confounding variables accounted for in design, conduct and analysis. Causation between investigated variables and LBSI measure is implied. |
| **Statistical analysis and reporting** | Low | P values and corrected Hedges’ g effect sizes reported. Multivariate analysis not reported in methods or results. |
