# Supplementary material for: Intrinsic variables associated with low back pain and lumbar spine injury in fast bowlers in cricket: a systematic review
Source: BMC Sports Sci Med Rehabil. 2023 Sep 20;15:114. doi: 10.1186/s13102-023-00732-1 (PMC10512628; doi:10.1186/s13102-023-00732-1)
Supplement: Supplementary file 4 — Additional file 4. Presents definitions for intrinsic variables that have been associated with low back pain and lumbar spine injury in fast bowlers. [file 13102_2023_732_MOESM4_ESM.docx]

**Additional File 4 Definitions for Intrinsic Variables**

**Age**

- Chronological age - the age of a person expressed in years as measured from birth to a given date.
- Skeletal age - the age of a person expressed in years that corresponds to the level of maturation of the skeleton, with this determination based on the presence of centres of bone formation as well as the dimension and structure of bones.

**Injury history**

- History of low back pain (LBP) - previous incidence of LBP determined by subjective examination or questionnaire.
- History of lumbar injury - previous incidence of lumbar spine injury determined by subjective examination, questionnaire, or sourcing of medical records and databases.

**Trunk and lumbar anatomical characteristics**

- Quadratus Lumborum asymmetry - percentage asymmetry derived by comparing bilateral Quadratus Lumborum Cross Sectional Area (CSA) or Volume as measured by MRI with participant positioned in supine.
- Multifidus CSA - Ultrasound measurement of the Lumbar Multifidus muscle CSA in square centimetres with participant positioned in prone with lumbar spine within 10° of horizontal.
- Abdominal muscle thickness - thickness in centimetres of the Internal Oblique, External Oblique, and Transversus Abdominus muscles measured by Ultrasound at the end of a normal quiet expiration with participant positioned in supine with the hips flexed to 45° and knees flexed to 90°.

**Lumbar spine radiological variables**

- Bone Mineral Content (BMC) - a measurement of bone mineral found in a specific area that is measured in grams.
- Vertebral body area - a measurement of the area in square centimetres of the vertebral body which is the thick, cylindrical, anterior and weight bearing portion of a vertebra that articulates with an adjacent vertebral body by an intervertebral disc.
- Bone Mineral Density (BMD) - a measurement of the amount of bone mineral contained in an area of bone that is derived by dividing the BMC by bone area and is normally expressed in grams/square centimetre.
- Lumbar disc degeneration - degenerative changes in the lumbar intervertebral disc related to a loss of disc height and disc hydration.
- Bone Marrow Oedema (BMO) - the presence of abnormal fluid signal seen within the bone marrow as detected by MRI.
- BMO Intensity Ratio - the ratio of the mean BMO intensity in the area with the highest signal intensity (in the pars interarticularis or pedicle region) divided by the mean BMO intensity in the largest possible area of the vertebral body. BMO intensity is measured by the elliptical region of interest tool in imaging software.

**Biomechanics of fast bowling technique**

- Ball release height - the vertical distance from the central core of the cricket ball to the ground at the instant that a cricket ball is released from the bowler’s hand during the fast bowling action.
- Ball release speed - the speed of release of a cricket ball at the instant that it is released from the bowler’s hand during the fast bowling action.
- Approach velocity - the mean horizontal velocity of a fast bowler during the run up (the bowler’s running approach to the wicket prior to the delivery stride) of the fast bowling action.
- Back foot contact (BFC) - the initial point when the back foot (the foot ipsilateral to the dominant arm) is in contact with the ground at the commencement of the delivery stride.
- Front foot contact (FFC) - the initial point when the front foot (the foot contralateral to the dominant arm) is in contact with the ground at the conclusion of the delivery stride.
- Delivery stride length - the calculated distance from back foot contact to front foot contact in the fast bowling action.
- Delivery stride alignment - the angle formed between back foot contact, front foot contact and a line representing the direction of ball travel.
- Shoulder alignment - the angle of the line represented by a line joining the acromion processes in the transverse plane in relation to the direction of ball travel. A front-on shoulder alignment is categorised if the shoulder alignment of greater than 200° is present in relation to the direction of ball travel, as per the graphical representation defining hip and shoulder alignment [169]
- Hip alignment - the angle of the line represented by a line joining the hip joints in the transverse plane in relation to the direction of ball travel, as per the graphical representation defining hip and shoulder alignment [169]


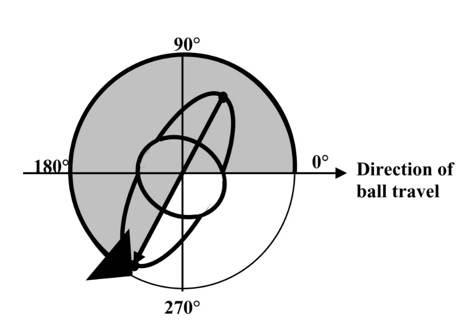


Graphical representation defining hip and shoulder alignment [169]

- Shoulder counter-rotation - the change between shoulder alignment at back foot contact and the minimum shoulder alignment during the delivery stride. This is calculated by subtracting the alignment of the shoulders when they are in their most side-on position (normally just prior to front foot contact), from the shoulder alignment at back foot contact.
- Hip counter rotation - the change between hip alignment at back foot contact and hip alignment during the delivery stride.
- Hip-shoulder separation - the angle that is calculated by subtracting the hip alignment angle from the shoulder alignment angle. A positive separation angle refers to the shoulders being in a more front-on alignment than the hip segment, a negative separation angle refers to the hip segment being more front-on than the shoulder segment.
- Front-on bowling technique - Mixed technique - a hip-shoulder separation angle equal to or greater than 30° at back foot contact, or shoulder counter rotation equal to or greater than 30°
- Side-on bowling technique – a shoulder segment angle less than 210° at back foot contact, a hip-shoulder separation angle less than 30° at back foot contact, and shoulder counter-rotation less than 30°
- Semi-open technique – a shoulder segment angle from 210 to 240° at back foot contact, a hip-shoulder separation angle less than 30° at back foot contact, and shoulder counter-rotation less than 30°.
- Mixed technique - a hip-shoulder separation angle equal to or greater than 30° at back foot contact, or shoulder counter rotation equal to or greater than 30°.
- Pelvis alignment – the angle of the pelvis in the transverse plane in a 3-D motion analysis model in relation to the direction of ball travel.
- Pelvis-shoulder separation - the angle that is calculated by subtracting the pelvis alignment angle from the shoulder alignment angle.
- Lateral flexion - the degree of lateral flexion (frontal plane motion) of either the trunk, thorax, thoracolumbar segment or lumbopelvic segment towards the non-bowling side.
- Rear hip angle - the degree of flexion or extension of the hip that is ipsilateral to the bowling arm and is normally measured during the back foot contact phase of the fast bowling action.
- Rear knee angle - the degree of knee flexion or extension of the knee that is ipsilateral to the bowling arm and is normally measured during the back foot contact phase of the fast bowling action.
- Front hip angle- the degree of flexion or extension of the hip that is contralateral to the bowling arm and is normally measured during the back foot contact phase of the fast bowling action.
- Front knee angle- the degree of flexion or extension of the knee that is contralateral to the bowling arm and is normally measured during the back foot contact phase of the fast bowling action.
- Flexion/extension - the degree of flexion/extension (sagittal plane motion) of the trunk, thorax, thoracolumbar segment or lumbopelvic segment
- Rotation - the degree of rotation (transverse plane motion) of either the trunk, thorax, thoracolumbar segment or lumbopelvic segment
- Front foot plant angle - the tilt orientation of the front foot at front foot contact that is represented by the angle between the global y-axis (the line pointing down the wicket) and a line joining a projection of the ankle and metatarsophalangeal joint centres onto a vertical global plane.
- Front leg plant angle - the tilt orientation of the front leg at front foot contact that is calculated by projecting the hip and ankle joint centres onto a vertical plane and represented by the angle between the downwards vertical and a line joining these two projected points.
- Ground Reaction Force - the force exerted by the ground on a body in contact with it.
- Tibial and sacrum acceleration - a proxy measurement for the impact forces experienced at the tibia and sacrum respectively, commonly measured with a segment mounted accelerometer.
- Peak resultant acceleration - the square root of the sum of squared accelerations along all three axes

**Physical testing**

- Sit-and-reach - a field test used to measure hamstring and low back flexibility, involving a participant sitting with feet approximately hip-wide against a testing box and slowly reaching forward as far as possible by sliding hands along a measuring board whilst knees remain extended.
- Straight leg raise - a test for hamstring flexibility which assesses the range of hip flexion whilst the knee is held in extension.
- Modified Thomas test - a test to assess hip extensibility and the presence of a hip flexion contracture.
- Bent knee fall-out - a test designed to measure hip and adductor muscle flexibility, involving a participant to allow their knee to ‘fallout’ into hip abduction and external rotation whilst in supine.
- Lunge test - a test involving a participant standing and lunging forward with the subtalar joint remaining in neutral and achieving maximal ankle dorsiflexion.
- Calf capacity - a test of calf endurance measured the maximum number of repeated maximal height single leg calf rises performed in knee extension.
- Single leg bridge capacity - a test of the ability to maintain a single leg bridge position in supine for the maximal length of time without lowering the hips, tilting the pelvis or arching the lumbar spine.
- Prone plank test - a test of the ability to maintain neutral spinal and pelvis alignment for the maximal length of time in a prone position with elbows flexed at 90° whilst resting on forearms and toes with the trunk raised off floor.
- Side plank test - a test of the ability to maintain neutral spinal and pelvis alignment for the maximal length of time in a side-lying position supported on one elbow flexed at 90°, whilst resting on one forearm and side of one foot with the trunk raised off floor.
- Biering-Sorensen test - a test for trunk extensor endurance assessing the maximal time the trunk can be actively held in a neutral position with a participant in prone and fixed to the testing table at the ankles, knees, and hips.
- Lumbar Reposition Error - the absolute difference between actual and participant replicated target positions in the assessment of lumbar proprioception impairment.
- Single leg decline squat - a test that involves a participant squatting to 90° of knee flexion with the trunk upright in in single leg stance on a board with 25° decline.
- Lumbopelvic stability test - a test that examines the ability to maintain neutral alignment of the lumbar spine and pelvis whilst performing staged lower limb movements.
- Star Excursion Balance – a test used to assess deficits in dynamic postural-control as assessed by a series of single-limb squats using the non-stance limb to reach maximally to touch a point along 1 of 8 designated lines on the ground.
